# Transcriptome Sequence Analysis of the Defense Responses of Resistant and Susceptible Cucumber Strains to *Podosphaera xanthii*

**DOI:** 10.3389/fpls.2022.872218

**Published:** 2022-05-12

**Authors:** Xiangnan Meng, Yongbo Yu, Tiefeng Song, Yang Yu, Na Cui, Zhangtong Ma, Lijie Chen, Haiyan Fan

**Affiliations:** ^1^College of Plant Protection, Shenyang Agricultural University, Shenyang, China; ^2^College of Bioscience and Biotechnology, Shenyang Agricultural University, Shenyang, China; ^3^Liaoning Academy of Agricultural Sciences, Shenyang, China; ^4^Key Laboratory of Protected Horticulture of Ministry of Education, Shenyang Agricultural University, Shenyang, China

**Keywords:** *Cucumis sativus* L., *Podosphaera xanthii*, transcriptome, resistance gene, initial stage control

## Abstract

Powdery mildew (PM) caused by *Podosphaera xanthii* poses a continuous threat to the performance and yield of the cucumber (*Cucumis sativus* L.). Control in the initial stages of infection is particularly important. Here, we studied the differential physiological and transcriptomic changes between PM-resistant strain B21-a-2-1-2 and PM-susceptible strain B21-a-2-2-2 at the early stage of *P. xanthii* attack. When challenged with *P. xanthii*, the tolerant line can postpone the formation of the pathogen primary germ. Comparative transcriptomic analysis suggested that DEGs related to the cell wall and to pathogen and hormone responses were similar enriched in both cucumber lines under *P. xanthii* infection. Notably, the number of DEGs triggered by *P. xanthii* in B21-a-2-1-2 was quintuple that in B21-a-2-2-2, revealing that the success of defense of resistant cucumber is due to rapidly mobilizing multiple responses. The unique responses detected were genes related to SA signaling, MAPK signaling, and Dof and WRKY transcription factors. Furthermore, 5 *P. xanthii* -inducible hub genes were identified, including *GLPK*, *ILK1*, *EIN2*, *BCDH*β*1*, and *RGGA*, which are considered to be key candidate genes for disease control. This study combined multiple analytical approaches to capture potential molecular players and will provide key resources for developing cucumber cultivars resistant to pathogen stress.

## Introduction

Cucumber (*Cucumis sativus*), a species of plant of the Cucurbitaceae family, is a key economic crop cultivated in protected horticultural areas, and it plays an indispensable role in the vegetable industry. However, with increasing planting density and continuous cropping, cucumbers are becoming more susceptible to various pathogens, especially powdery mildew (PM). PM is widely distributed, has a short incubation period, and is transmitted rapidly, leading to frequent epidemics and making PM an important global disease (Chen et al., 2021).

PM in cucumbers is mainly caused by *Golovinomyces chicoracearum* (formerly *Erysiphe cichoracearum*) and *Podosphaera xanthii* (formerly *Sphaerotheca fuliginea*), of which *P. xanthii* is more common (Gao et al., 2020). Both pathogens belong to the subphylum Ascomycota and are generally distinguished by the types of conidia and their germination methods (Ren, 2011). When the conidia contact a surface on the host, PM fungi adsorb to the host and penetrate into their stratum corneum and cell walls. Every PM fungus produces 1 or 2 germination tubes, which swell at the top to form an appressorium, which is the main organ mediating host invasion. PM fungi can also invade the cell walls of the host, mainly relying on enzymes and mechanical power. After invading the cell walls, the PM fungi continue to produce haustoria to invade the host cell membranes, to obtain nutrients, and then to enter the host cells themselves (Hückelhoven, 2005).

These PM pathogens are living vegetative fungi that are transmitted by air and that mainly infect cucumber leaves. When the fungi encounter cucumber leaves, they initially affect respiration and photosynthesis by forming a layer of white powder on the surface, invasiveness into the plant cells which results in the weakening of growth and even the death of the plant (Bi et al., 2016). Even this initial surface-level infestation, then, reduces yields and can cause substantial economic losses, critically impacting the cucumber industry. Hence, control of PM at its initial stages is particularly important. Understanding the process of infection by PM fungi in cucumbers and the molecular mechanisms of interaction between cucumbers and PM fungi can provide a scientific basis for controlling PM. These efforts will contribute to increasing cucumber performance and yield.

Cucumber plants have multiple mechanisms to resist fungal infections, and the interactions between cucumbers and PM fungi are strongly influenced by resistance genes. While previous studies have confirmed that cucumber PM resistance is controlled by multiple resistance loci, most of these studies have been limited to quantitative trait locus mapping (Shen, 2009; Yu, 2015; Wang et al., 2020; Liu et al., 2021). At present, only a few recessive resistance genes, mainly including *Mildew Resistance Locus O* (*MLO*) and *NBS-LRR*-type resistance gene families, have been successfully cloned and validated (Nie et al., 2015; Shi, 2016; Wang et al., 2021). Therefore, mining for additional PM resistance-related genes is of great significance for controlling the occurrence of PM and increasing cucumber yield.

In this study, RNA-sequencing (RNA-seq) analysis was performed on the cotyledons of a PM-resistant cucumber strain (B21-a-2-1-2) and a PM-susceptible cucumber strain (B21-a-2-2-2) that had been infected with *P. xanthii*, so as to identify differentially expressed genes (DEGs). We investigated biology processes and pathways that are enriched in these DEGs and that potentially contribute to PM resistance. Additionally, based on weighted gene co-expression correlation network analysis (WGCNA), 5 *P. xanthii*-inducible hub genes were detected. This study was designed to lay a theoretical foundation for further research on the regulatory mechanisms of cucumber PM resistance.

## Materials and Methods

### Plant Growth and Inocula

The cucumber tested in this study were the two sister lines, PM-resistant strain B21-a-2-1-2 and PM-susceptible strain B21-a-2-2-2, which are provided by Liaoning Academic of Agricultural Science. The two lines were selected from a segregated population originated from four generation selfing of a South Korea cultivar. They are different in resistance to PM, but alike in the plant type, commodity characteristics, tolerance to other stresses, and so on.

Cucumbers were cultivated in a 26°C greenhouse with 16 h light/8 h dark cycles. 9-day-old cotyledons of cucumber lines were inoculated with *P. xanthii* (10^5^ conidia mL^–1^) by uniform spray as described previously (Ren, 2011). The cotyledons were harvested at seven time points [0, 6, 12, 24, 48, and 96 h post-inoculation (hpi) and 7 days post-inoculation (dpi)] and the euphylla were harvested 7 dpi. The tissues were quick-frozen in liquid nitrogen and stored at –80°C for later use.

### Investigation of Coomassie Brilliant Blue Staining and Disease Index

Coomassie brilliant blue staining was used to detect the changes of *P. xanthii* in inoculated cucumber cotyledons. Firstly, cucumber cotyledons were soaked in decolorizing solution containing trichloroacetic acid, absolute ethanol and chloroform (0.225:150:50, w/v/v), and incubated at 70°C for more than 30 min until the leaves were white and transparent. Then, they were placed in staining solution containing trichloroacetic acid, Coomassie brilliant blue, methanol and ddH_2_O (0.225:0.9:150:150, w/w/v/v) for 5 min. The stained tissues were rinsed with distilled water to remove the dye liquor and observed under an optical microscope.

The disease index was determined to quantify the incidence of *P. xanthii* on inoculated cucumber cotyledons and euphylla. The disease index survey procedure and the grading criteria were used as described by Meng et al. (2018).

### RNA Isolation and Sequencing

RNA was extracted according to the method described in the RNAprep Pure Plant Kit (Tiangen, Beijing, China). The purity and concentration of RNA were detected with a BioDrop μLite spectrophotometer (BioDrop in Cambridge CB4 OFJ England). The integrity of the RNA was assessed by 1% agarose gel electrophoresis. RNA samples extracted from B21-a-2-1-2 and B21-a-2-2-2 cucumber cotyledons harvested at 0 and 6 hpi were sequenced on the Illumina Hiseq platform (Personalgene, Nanjing, China). Equal volumes of the RNA from the three biological replicate samples at each time point were mixed prior to sequencing.

Clean data was obtained by filtering out the joints and low-quality reads of raw data. Clean data was mapped to the cucumber genome database^1^ *via* HISAT2 (an updated version of TopHat2).^2^ The mapping was considered successful when the mismatches between the default reads and the reference genome sequences were within 2. Htseq was used to calculate the read count value mapped to each gene, and this value was considered the original level of expression of the gene. The expression was then standardized as fragments per kilobase million (FPKM). DESeq was used for differential analysis of gene expression. The screening conditions of differentially expressed genes (DEGs) were | log2FoldChange| > 1 and *p*-value < 0.05.

### Functional Annotation

For systematic analysis of gene functions, all DEGs were mapped according to the Gene Ontology (GO)^3^ and Kyoto Encyclopedia of Genes and Genomes (KEGG)^4^ internet utilities. Following GO and KEGG analysis, Mapman 3.6.0RC1^5^ was used to trace the transcriptome changes regarding cucumber- *P. xanthii* interactions (Thimm et al., 2004). A *p*-value < 0.05 was considered as the threshold for significant enrichment.

### Module Construction and Identification of Hub Genes

The WGCNA package 1.70-3 in R software 4.04 was used to screen for key modules related to cucumber defense against *P. xanthii* infection (Langfelder and Horvath, 2008). After removing the outliers (FPKM values > 0), 18,035 genes were selected for further analysis. We set a weighted correlation threshold of > 0.85, the optimal power at 22, the minimal module size at 30 and the branch merge cut height at 0.25. Other parameters were maintained at their default settings. For exploring the key module gene functions, GO and KEGG enrichment analyses were also constructed. Subsequently, hub genes were identified on the basis of Module Membership (MM) > 0.98 and edge weight value > 0.5.

### qRT-PCR Validation

To verify the reproducibility of RNA-seq results, we selected 16 DEGs to analyze by qRT-PCR. RNA extraction and quality detection were carried out as described above. The first strand of cDNA was synthesized with the FastQuant RT Kit (Tiangen, Beijing, China). A LightCycler 480 (Roche Molecular Systems, CA, United States) was used to perform qRT-PCR using the SuperReal PreMix Plus Kit (Tiangen, Beijing, China). *CsActin* was used as an internal reference to normalize the data. The relative expression levels of genes were computed with the 2^–ΔΔ*Ct*^ quantitative analysis method. The primers used in this validation are shown in Supplementary Table 1.

### Accession Code

All raw sequencing reads were deposited to the NCBI Sequence Read Archive under the project ID PRJNA816625.

## Results

### *Podosphaera xanthii* Inoculation and Plant Responses

We inoculated the cotyledons and euphylla of PM-resistant (B21-a-2-1-2) and PM-susceptible (B21-a-2-2-2) cucumbers with *P. xanthii* to test varieties in phenotypes and lesions. At 7 d after inoculation, the *P. xanthii* infection on B21-a-2-2-2 leaves was clearly more serious than on B21-a-2-1-2, and investigation using the disease index (DI) yielded results that were compatible with this conclusion (Supplementary Figure 1). Furthermore, we also detected the expression level of *P. xanthii* by qRT-PCR and found that its expression level in B21-a-2-1-2 was remarkably lower than that in B21-a-2-2-2. These results supported the reliability of the experimental materials.

**FIGURE 1:**
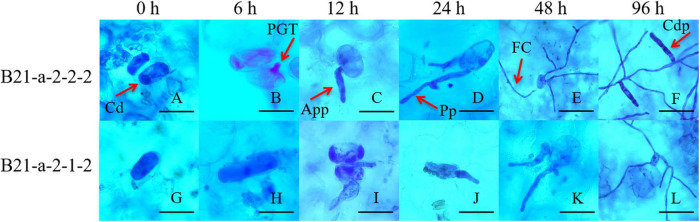
Process of infection of *P. xanthii* of the PM-susceptible cucumber strain B21-a-2-2-2 and the PM-resistant cucumber strain B21-a-2-1-2. Cd, conidia; PGT, primary germ tube; App, appressorium; Pp, penetration peg; FC, fungal colony; Cdp, conidiophore. The bar in panels A through D and G through K is 20 μm, and the bar in panels E through F and L is 60 μm.

We also inoculated the cotyledons of B21-a-2-1-2 and B21-a-2-2-2 cucumbers with *P. xanthii*, and carried out Coomassie brilliant blue staining at 0, 6, 12, 24, 48, and 96 hpi to observe the process of *P. xanthii* infection. As shown in Figure 1, for B21-a-2-2-2 cucumbers, the primary germ tube (PGT), appressorium (App), penetration peg (Pp), fungal colony (FC), conidiophore (Cdp) were successively observed at 6, 12, 24, 48, and 96 hpi, and spores were found to be able to complete their asexual growth cycle.

However, for B21-a-2-1-2 cucumbers, only spores could be observed at 6 hpi, and spores began to germinate and to form a small amount of PGT at 12 hpi. A few App were formed, and most of the spores remained in the PGT stage at 24 hpi. Most of the spores stopped germinating and remained in the App stage, and only a few spores formed Pp at 48 hpi. FC was formed at 96 hpi, but Cdp was not seen in the visual field. Together, these observations indicated that the spores of *P. xanthii* could not complete their asexual growth cycle in the resistant strain B21-a-2-1-2. These results showed that the process of infection of *P. xanthii* in the B21-a-2-1-2 strain showed a substantial lag compared with the B21-a-2-2-2 strain, and the difference in the process was apparent by 6 hpi. Therefore, we used 0 and 6 hpi samples for the following studies.

### RNA-Seq Analysis and Mapping to the *Cucumis sativus* Reference Genome

To characterize the transcriptional response of cucumbers to *P. xanthii* inoculation, high-throughput sequencing was performed using RNA isolated from resistant (B21-a-2-1-2) and susceptible (B21-a-2-2-2) tissues at 0 and 6 hpi. Three biological replicates were performed for each sample. A total of 41.6–66.0 million raw reads were yielded from the 12 transcriptome libraries (Table 1). After trimming of adapter and low-quality sequences, the numbers of clean reads ranged from 39.8 to 66.0 million, and the proportions of clean reads of samples (Q30) were 93.9–94.5%. The correlation coefficients of the expression levels of each sample are shown in Supplementary Figure 2, and principal components analysis is exhibited in Supplementary Figure 3 and Supplementary Table 2. These parameters indicated that the sequences used in the following analyses were of high quality. Of the clean reads, 95.2–96.1% were uniquely aligned to the *C. sativus* Gy14 genome v2.

**TABLE 1:** Filtering and assessing of RNA-seq data.

Sample	Raw reads	Clean reads	Ratio of clean reads (%)	Q30 (%)	Total mapped (%)
S0h1	51,109,306	47,037,856	92.03	94.45	96.13
S0h2	60,633,286	55,716,870	91.89	94.43	95.78
S0h3	55,672,804	51,344,320	92.22	94.37	95.20
R0h1	49,770,046	45,680,892	91.78	94.32	96.02
R0h2	54,049,194	49,424,606	91.44	94.44	95.60
R0h3	45,092,538	41,566,714	92.18	93.92	95.86
S6h1	52,827,930	48,735,520	92.25	94.03	95.67
S6h2	43,146,846	39,801,022	92.24	94.05	95.59
S6h3	71,216,142	65,968,022	92.63	94.37	96.06
R6h1	57,497,840	52,693,302	91.64	94.48	95.67
R6h2	47,430,404	43,620,472	91.96	94.53	95.51
R6h3	56,898,606	52,233,034	91.80	94.46	95.68

**FIGURE 2:**
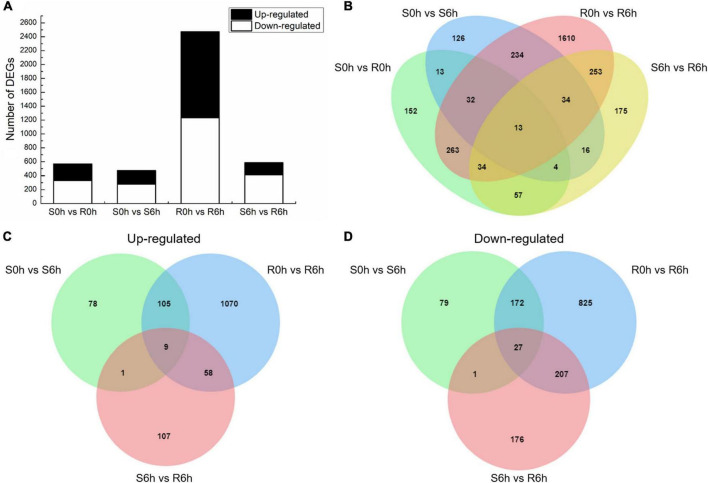
DEGs between the PM-susceptible strain B21-a-2-2-2 (S) and PM-resistant strain B21-a-2-1-2 (R) of cucumber in response to *P. xanthii*. **(A)** Numbers of DEGs obtained in each comparison. **(B)** Overlap of DEGs in each comparison. **(C)** Venn diagram of genes up-regulated in S0h vs. S6h, R0h vs. R6h, and S6h vs. R6h comparisons. **(D)** Venn diagram of genes down-regulated in S0h vs. S6h, R0h vs. R6h, and S6h vs. R6h comparisons.

Using the criteria of | log2 FC| > 1 and *q*-value < 0.05, we obtained a total of 4,099 DEGs by comparing RNA expression in the susceptible to the resistant strains at 0 hpi (S0h vs. R0h), the susceptible and resistant strains at 6 hpi (S6h vs. R6h), the resistant strain at 0 hpi to the resistant strain at 6 hpi (R0h vs. R6h), and the sensitive strain at 0 hpi to the sensitive strain at 6 hpi (S0h vs. S6h) (Figure 2 and Supplementary Figure 4). In total, 568 DEGs were identified in the S0h vs. R0h comparison, of which 236 were up-regulated and 332 were down-regulated (Supplementary Table 3). Upon *P. xanthii* inoculation, 472 DEGs (193 up-regulated and 279 down-regulated) were identified in the susceptible line (Supplementary Table 4), while 2,473 DEGs (1,242 up-regulated and 1,231 down-regulated) were identified in the resistant line (Supplementary Table 5). Among these DEGs, 313 transcripts were shared between both lines, while 2160 and 159 genes were specifically expressed in the resistant and susceptible line, respectively (Figure 2B). These data indicated that the resistant line mobilized more unique responses to fight against *P. xanthii*.

By comparing S0h vs. S6h, R0h vs. R6h and S6h vs. R6h, 36 transcripts were identified as the same expression trend DEGs, of which 9 were up-regulated and 27 were down-regulated (Figures 2C,D). The up-regulated transcripts included E3 ubiquitin-protein ligase (*RNF115*), caffeoyl shikimate esterase (*CSE*), peroxidase 2-like (*POX 2*-like), glycine-rich cell wall structural protein 1.8 (*GRP 1.8*), calcium-dependent protein kinase 8-like (*CDPK 8*-like), major pollen allergen Ole e 6, and beta-amylase, and two genes encoding uncharacterized protein (Supplementary Table 6). Among these, *CSE* and *POX* both code for key enzymes in the lignin biosynthesis pathway, and *GRP* codes for a plant cell wall structural protein (Mceldoon et al., 2012; Vanholme et al., 2013; Zhao et al., 2020). Lignin is component of the plant secondary cell wall, therefore indicating that resistant plants perform more cell wall component alterations.

### Functional Category Enrichment of Differentially Expressed Genes

**FIGURE 3:**
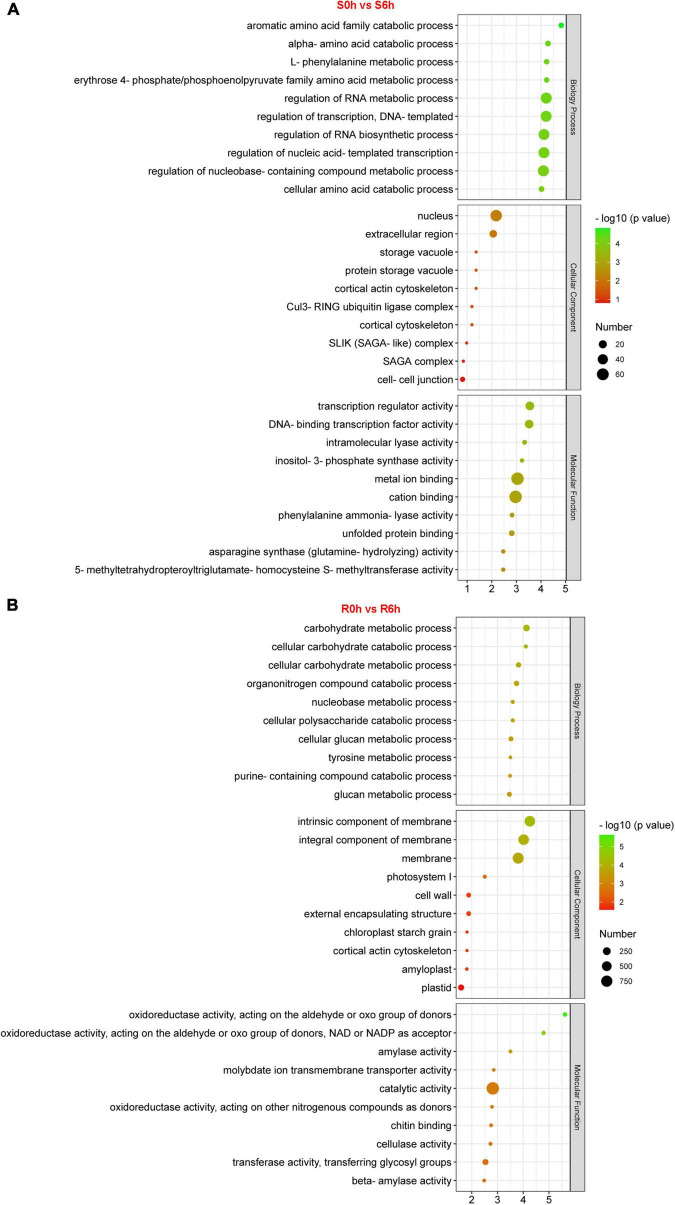
GO enrichment analysis of DEGs in PM-susceptible cucumber B21-a-2-2-2 **(A)** and PM-resistant cucumber B21-a-2-1-2 **(B)** subjected to *P. xanthii*. The results from the biological process, cellular component and molecular function categories are summarized.

GO, KEGG and Mapman analyses were performed to capture key biology processes or pathways involved in defense against *P. xanthii* in the resistant and susceptible cucumber. Through analysis of GO terms, 343 and 1,886 DEGs were functionally annotated in S0h vs. S6h and R0h vs. R6h, respectively (Figure 3 and Supplementary Figures 5–10). Biology processes such as regulation of RNA metabolic and biosynthetic process, transcription and nucleobase-containing compound metabolic process were obviously enriched in S0h vs. S6h. In R0h vs. R6h, carbohydrate metabolic process was the largest biology process gene ontology. *P. xanthii* clearly affected cell extracellular region, especially the cell wall, in both lines, as evidenced by GO terms in the cellular component category. Additionally, GO terms in molecular function for each comparison were quite different. Transcription regulator activity, cation binding and metal ion binding are the three most enriched GO terms in S0h vs. S6h, while catalytic activity comprised the majority of terms in R0h vs. R6h.

**FIGURE 4:**
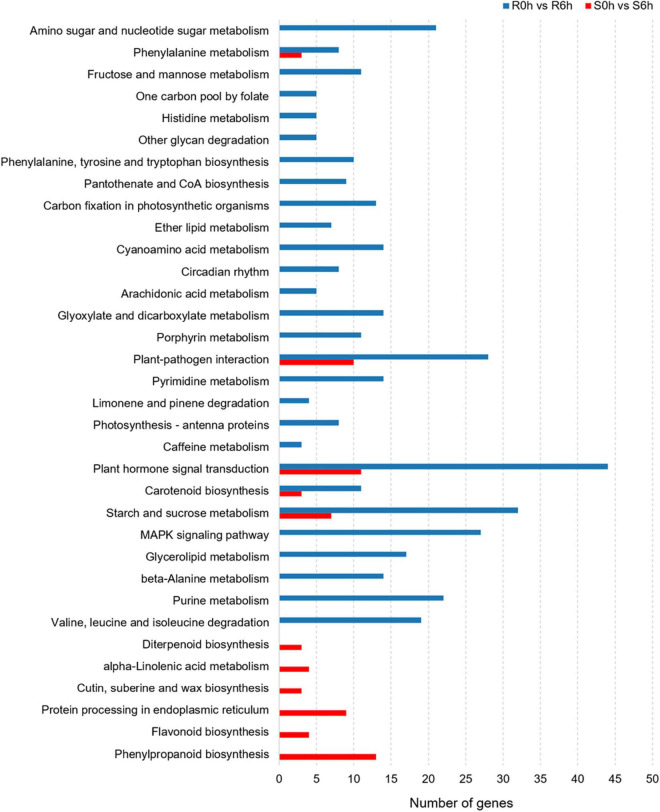
KEGG pathway analysis of DEGs in PM-susceptible cucumber strain B21-a-2-2-2 and PM-resistant cucumber strain B21-a-2-1-2 subjected to infection with *P. xanthii*.

Considering KEGG classifications, the *P. xanthii*-resistance mechanism of the resistant line was different from that of susceptible line. In S0h vs. S6h, pathways such as plant hormone signal transduction, protein processing in endoplasmic reticulum, phenylpropanoid biosynthesis and plant-pathogen interaction were significantly enriched. In R0h vs. R6h, the function classes of plant hormone signal transduction, starch and sucrose metabolism, MAPK signaling pathway and plant-pathogen interaction were obviously enriched (Figure 4).

**FIGURE 5:**
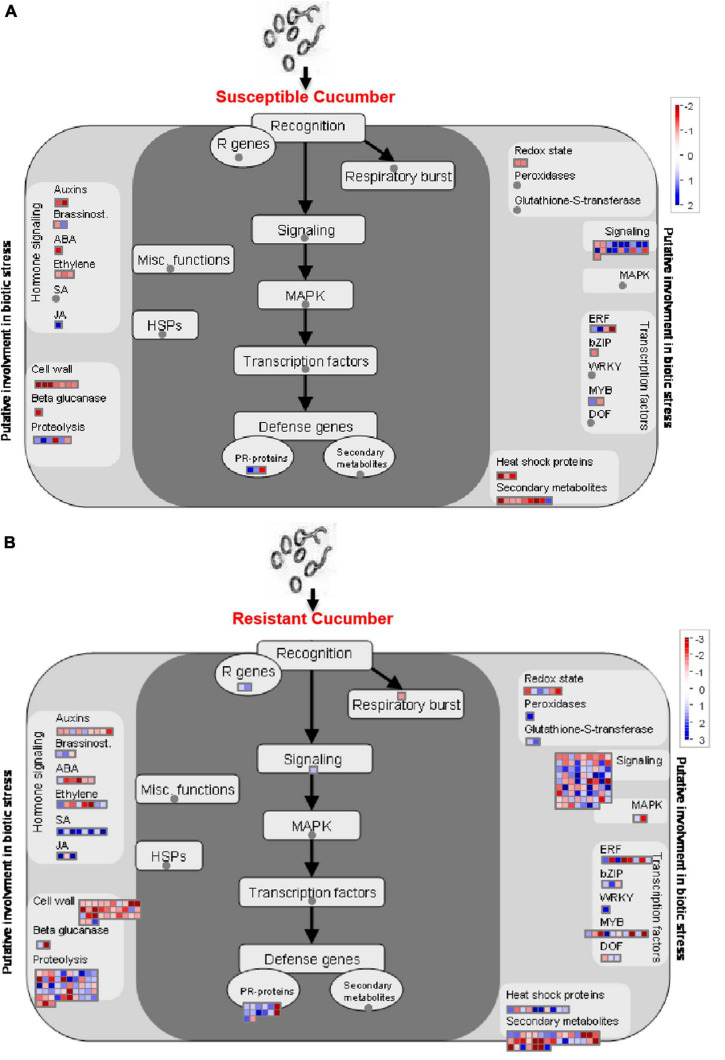
Mapman analysis of DEGs in PM-susceptible cucumber strain B21-a-2-2-2 **(A)** and PM-susceptible cucumber strain B21-a-2-2-2 **(B)** subjected to infection with *P. xanthii*. The blue and red dots represent up- and down-regulated genes.

By using Mapman analysis, we found a number of pathways involved in the regulation of defense against *P. xanthii* in the resistant cucumber that were absent in the susceptible line (Figure 5). Most defense-related DEGs were up-regulated in R0h vs. R6h, while these DEGs were totally absent in S0h vs. S6h. DEGs related to salicylic acid (SA) signaling, MAPK signaling and gene transcription regulated by Dof and WRKY transcription factors were detected in R0h vs. R6h, but not present in S0h vs. S6h, revealing that these pathways likely affect cucumber resistance to *P. xanthii.* In addition, the resistant cucumber also exhibited obvious changes to transcripts related to cell wall, proteolysis, signaling and secondary metabolites. These pathways have been reported to be involved in responses to abiotic and biotic stresses in plants. Taken together, DEGs related to cell wall component, signaling and gene transcription regulation are collectively involved in regulating the defense response in cucumber, resulting in different degrees of resistance in different plant materials.

### Screening for Key Modules Related to *Podosphaera xanthii* Defense in Cucumber

**FIGURE 6:**
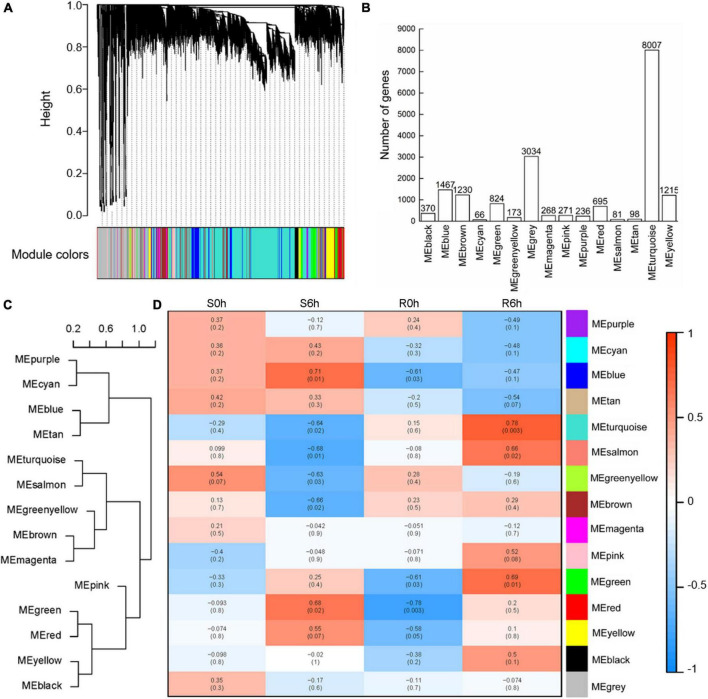
WGCNA analysis of key modules related to *P. xanthii* defense in cucumber. **(A)** Clustering dendrogram of co-expression modules. **(B)** The number of genes detected in each module. **(C)** Heat maps of gene expression in each module. Cluster tree **(C)** and heat maps **(D)** of gene expression in each module.

To capture cohorts of genes associated with cucumber defenses against *P. xanthii*, a total of 18,035 genes were selected to conduct WGCNA after filtration of the outliers. By using the WGCNA platform, we obtained 15 different modules (decorated with different colors) based on similarities of expression patterns (Figure 6A). Among them, the largest module (turquoise) comprised 8,007 genes, whereas the smallest module (cyan) comprised only 66 genes (Figure 6B). These modules were clustered into two clades according to module eigengenes (Figure 6C). The correlation coefficients between each sample and module eigengenes ranged from –0.78 to 0.78 (Figure 6D). It is worth noting that the turquoise module was obviously positive in the resistant strain at 6 hpi (*r* = 0.78, *p*-value = 0.003) and negative in the susceptible strain at 6 hpi (*r* = –0.64, *p*-value = 0.02); the blue module was positive in the susceptible strain at 6 hpi (*r* = 0.71, *p*-value = 0.01), while the salmon module was negative in the susceptible strain at 6 hpi (*r* = –0.68, *p*-value = 0.01); the red module was negative in the resistant strain at 0 hpi (*r* = –0.78, *p*-value = 0.003) and positive in the susceptible strain at 6 hpi (*r* = 0.68, *p*-value = 0.02) (Figure 6C). These data indicated that the turquoise module was most closely correlated with *P. xanthii* resistance.

**FIGURE 7:**
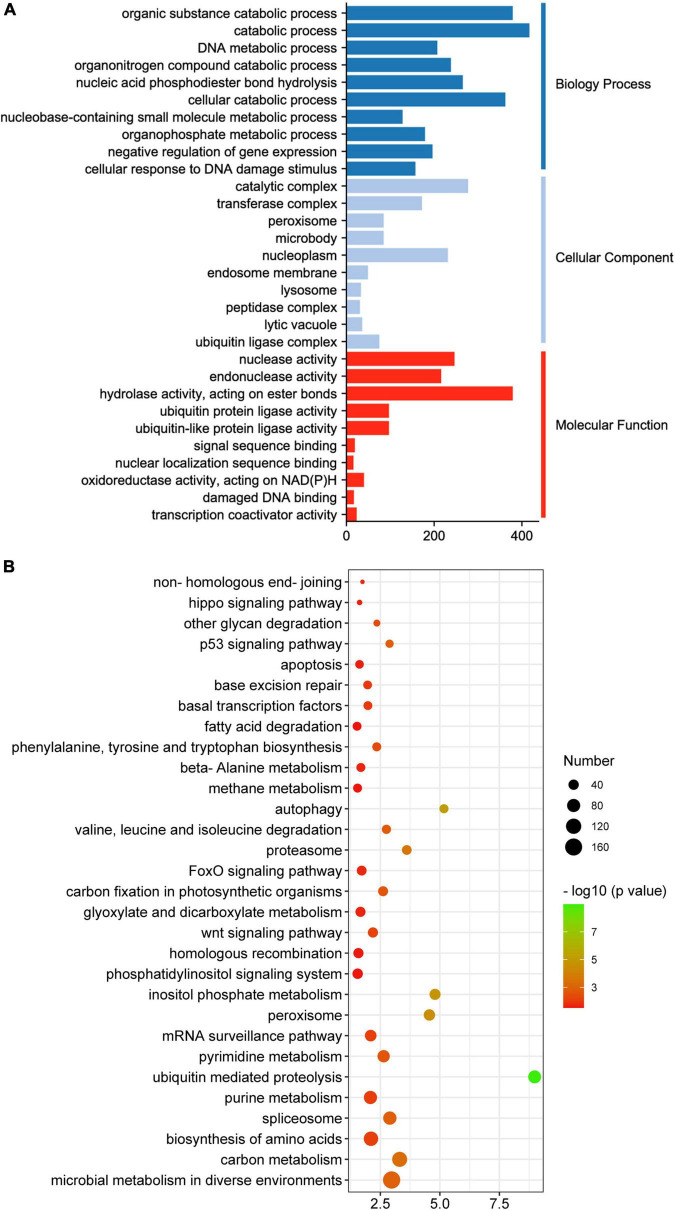
GO **(A)** and KEGG **(B)** analysis of genes in the turquoise module.

Upon GO enrichment analysis, 564 significantly enriched GO terms were identified in the turquoise module, with 401 of them in biological process, 72 in cellular component and 91 in molecular function. The most prevalent biological process function GO terms were catabolic process (417 genes), organic substance catabolic process (379 genes) and cellular catabolic process (362 genes). Of the cellular component group, catalytic complex (277 genes), nucleoplasm (231 genes) and transferase complex (172 genes) represented the three largest GO terms. Under the classification of molecular function group, hydrolase activity (379 genes), nuclease activity (246 genes) and endonuclease activity (216 genes) were the mostly abundant (Figure 7A). Based on KEGG pathway analysis, the turquoise module was found to mainly participate in microbial metabolism in diverse environments, carbon metabolism, biosynthesis of amino acids, spliceosome and purine metabolism (Figure 7B).

### Identification of Hub Genes Within Key Modules

In order to find hub genes in the response to *P. xanthii* stress, we constructed a gene correlation network from the turquoise module. Based on the criteria of MM > 0.98 and edge weight value > 0.5, 32 genes were regarded as hub genes (Table 2). Of the 32 hub genes, 5 genes were found to participate in responses to stimuli. These genes included CsGy7G018660 (glycerol kinase, *GLPK*), CsGy5G023580 (integrin-linked protein kinase 1, *ILK1*), CsGy1G028370 (2-oxoisovalerate dehydrogenase subunit beta 1, *BCDH*β*1*), CsGy6G023850 (ethylene-insensitive protein 2, *EIN2*), and CsGy6G008390 (RGG repeats nuclear RNA binding protein A, *RGGA*).

**TABLE 2:** List of hub genes identified in the turquoise module.

Gene ID	MM	Gene symbol	Function
CsGy7G018660	0.992428363	Glycerol kinase (GLPK)	Required for resistance to bacteria and pathogenic fungus
CsGy4G008900	0.991167397	D-xylose-proton symporter-like 2	Sugar transport
CsGy5G007990	0.98906476	PHD finger protein ALFIN-LIKE 2	Chromatin organization
CsGy5G023580	0.988856205	Integrin-linked protein kinase 1 (ILK1)	Functions as a link between plant defense pathways, stress responses and potassium homeostasis
CsGy1G007230	0.9875807	Dihydroorotate dehydrogenase family protein	Beta-alanine biosynthetic process
CsGy7G011040	0.986838679	Calcium-dependent protein kinase 11 (CDPK11)	Regulate the calcium-mediated abscisic acid (ABA) signaling pathway
CsGy3G008770	0.985873481	Ubiquitin-conjugating enzyme E2	Ubiquitin-dependent protein catabolic process
CsGy6G030310	0.985834795	Probable Xaa-Pro aminopeptidase P	Catalyzes the removal of a penultimate prolyl residue from the N-termini of peptides
CsGy1G020430	0.985767279	Transcription factor bHLH35	Regulation of transcription
CsGy3G026100	0.985691324	Hypothetical protein	
CsGy7G011190	0.9851292	Uncharacterized protein	
CsGy4G007480	0.9850624	Cationic amino acid transporter 8 (CAT8)	Amino acid transport
CsGy1G028370	0.984692108	2-Oxoisovalerate dehydrogenase subunit beta 1 (BCDH β1)	Response to nutrient
CsGy3G009460	0.984409171		
CsGy3G017100	0.983794852	Signal peptide peptidase-like 1(SPPL1)	Signal peptide processing, membrane protein proteolysis
CsGy5G012190	0.983413496	Protein NRT1/PTR FAMILY 8.3 (NPF8.3)	Peptide, high affinity, low capacity, and histidine transporter
CsGy1G017480	0.983368664	Hypothetical protein	
CsGy2G016990	0.98326016	Uncharacterized protein	
CsGy5G022510	0.98296069	Uncharacterized protein	
CsGy3G029810	0.982931934	PRA1 family protein	Secretory and endocytic intracellular trafficking in the endosomal/prevacuolar compartments
CsGy2G008200	0.982797143	Phospholipase A(1) LCAT3	Lipid metabolic process
CsGy3G012940	0.982593503	1,2-Dihydroxy-3-keto-5-methylthiopentene dioxygenase	Methionine metabolic process
CsGy7G008770	0.982104612	Uncharacterized protein	
CsGy3G002200	0.981597838	GATA transcription factor 26	Regulation of transcription
CsGy6G005750	0.981397749	Uncharacterized protein	
CsGy6G008390	0.981093123	RGG repeats nuclear RNA binding protein A (RGGA)	Involved in resistance to salt and drought stresses
CsGy3G030790	0.980991378	DNA-binding protein S1FA	Regulation of transcription
CsGy7G020980	0.980785958	Probable phospholipid-transporting ATPase 4 (ALA4)	Phospholipids transport
CsGy6G034530	0.980455722	Transcription factor VOZ1	Regulation of transcription
CsGy6G023850	0.980416952	Ethylene-insensitive protein 2 (EIN2)	Involved in various processes including development, plant defense, senescence, nucleotide sugar flux, and tropisms
CsGy3G026520	0.980378361	GDSL esterase/lipase 5 (GLIP5)	Lipid catabolic process
CsGy1G019590	0.980365209	Hypothetical protein	

Four genes were found to participate in transcriptional regulation, including CsGy1G020430 (transcription factor bHLH35), CsGy3G002200 (GATA transcription factor 26), CsGy3G030790 (DNA-binding protein S1FA), and CsGy6G034530 (transcription factor VOZ1). Four genes were found to participate in molecular transport, including CsGy4G008900 (D-xylose-proton symporter-like 2, sugar transporter), CsGy4G007480 (cationic amino acid transporter 8, amino acid transporter), CsGy5G012190 (protein NRT1/PTR FAMILY 8.3, peptide, high affinity, low capacity, and histidine transporter), and CsGy7G020980 (probable phospholipid-transporting ATPase 4, phospholipids transporter). Two of the hub genes were found to participate in lipid metabolic process, including CsGy2G008200 (phospholipase A(1) LCAT3) and CsGy3G026520 (GDSL esterase/lipase 5). In addition, CsGy3G008770 (ubiquitin-conjugating enzyme E2) was annotated to participate in protein ubiquitination and CsGy7G011040 (calcium-dependent protein kinase 11, *CDPK11*) in abscisic acid (ABA) signaling pathways. Function annotation thus demonstrated that these hub genes were mainly related to defense responses, transcriptional regulation, molecular transport and lipid metabolic process.

### Validation of Gene Expression Profiles by qRT-PCR

**FIGURE 8:**
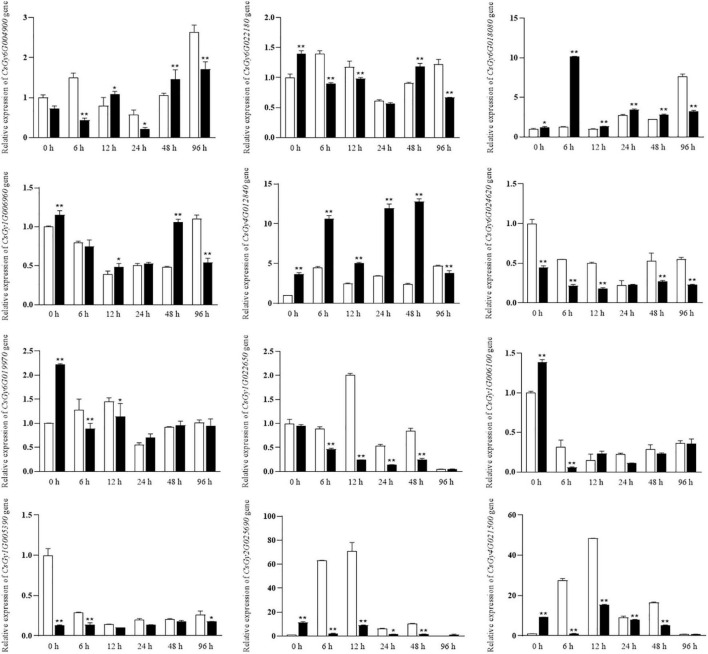
Expression analysis of select DEGs at 0, 6, 12, 24, 48, and 96 hpi using the 2^– ΔΔ*Ct*^ method. Data are means ± SD of three biological replicates per variety. Significance was determined by Duncan’s multiple range test, and is represented by *P ≤ 0.05 and **P ≤ 0.01.

To validate the reliability of the RNA-seq data and to analyze the expression levels of stress-responsive genes, 16 DEGs were selected for qRT-PCR assays. We conducted experiments at six time points (0, 6, 12, 24, 48, and 96 hpi) using the B21-a-2-1-2 and B21-a-2-2-2 strains (Figure 8). Most DEGs expression levels were consistent with the RNA-seq results, indicating the reproducibility of the data. Notably, the expression level of more DEGs underwent a striking change in B21-a-2-1-2 than in B21-a-2-2-2 in the early stages of infection.

## Discussion

This study was designed to capture key genome-related parameters related to the resistance of cucumber to *P. xanthii*. *P. xanthii* does not infect cucumber fruit directly. Instead, they weaken growth and impact fruit yield by invading cucumber leaves, reducing photosynthesis and increasing respiration and transpiration. Hence, control at the initial stages of infection is clearly optimal in order to reduce yield loss. Therefore, we selected 6 hpi for RNA-seq analysis, because that is a critical point when spores germinate on resistant and susceptible cucumber lines. In this study, the resistant line had comparatively more DEGs than did the susceptible line under *P. xanthii* invasion. This finding was similar to those found in previous transcriptomic studies in sorghum, sugarcane and wheat, in which resistant lines induced expression of more DEGs than did susceptible lines under biotic stress (Kiani and Szczepaniec, 2018; De Mello et al., 2020; Kiani et al., 2021).

Plants have evolved complex defense mechanisms to ward off invading pathogens. The cell wall, which covers the outermost surfaces of the plant, offers a frontline barrier against pathogen invasion. The plant cell wall is a complex and dynamic network that mainly consists of phenolic compounds (lignin), matrix polysaccharides (hemi-cellulose and pectin), structural polysaccharides (cellulose) and protein (Sandhu et al., 2009). Numerous studies have focused on ways in which the cell wall differs in resistant and susceptible plant hosts (Meng et al., 2016; Otulak-Kozieł et al., 2020; Kozieł et al., 2021). In our dataset, cell wall-related genes, such as *CSL* (cellulose synthase, CsGy6G024620, and CsGy2G018180), *FLA* (fasciclin-like arabinogalactan protein, CsGy7G007790), *GH* (glycosyl hydrolase, CsGy6G021790), *EXP* (expansin, CsGy5G023420, CsGy4G014170, and CsGy5G028430), *PGX* (polygalacturonase involved in expansion, CsGy2G017040, and CsGy5G022100), *XTH* (xyloglucan endotransglucosylase/hydrolase, CsGy6G022130, CsGy3G038470, CsGy1G019960, and CsGy1G019970), *PE* (pectinesterase, CsGy1G026850, CsGy2G012280, CsGy3G000030, CsGy3G026590, and CsGy4G005120), *PAE* (pectin acetylesterase, CsGy1G025090), *AP* (aspartyl protease, CsGy5G027100) and *BGAL* (beta-galactosidase, CsGy6G030970) tended to be down-regulated in the resistant line under *P. xanthii* stress.

It would seem reasonable to predict that cell wall-related genes confer resistance by augmenting wall strength; conversely, our data suggest that down-regulation of these cell wall-related genes confers resistance against *P. xanthii* in cucumber, perhaps owing to an activation of the plant immune response. This suggestion is in line with a previously published test of cell wall mutants in *Arabidopsis*, in which 81.6% of cell wall mutants were found to enhance resistance to necrotrophic and vascular pathogens (Molina et al., 2021). Lignin has been previously reported to provide a protective barrier upon infection and resistance against pathogens (Miedes et al., 2014; Wang X. et al., 2018). Infection by *P. xanthii* also markedly induced expression of lignin biosynthetic genes *CSE* (CsGy1G010260) and *POX* (CsGy4G012840) in the resistant line. Hence, these cell wall-related genes may either negatively or positively impact the resistance against pathogen infection, depending on the specific defense mechanism activated.

Plant hormone-mediated signaling pathways play an important role in plant defense responses to biotic stress. For example, JA- and ET- mediated signaling pathways contribute to plant resistance to necrotrophic pathogens, while SA-mediated signaling pathway is involved in plant resistance to biotrophic pathogens (Chen et al., 2010). Indeed, application of exogenous SA can induce cucumber resistance to *P. xanthii* (Mo, 2005). In our experiment, two salicylic acid glucosyltransferase 1 genes (*SGT1*, CsGy3G025850, and CsGy3G025860) were detected up-expressed only in the resistant cucumber in response to *P. xanthii* attack. SGT also known as uridine diphosphate (UDP)-glucosyltransferase (UGT), which converts SA into SA 2-O-β-D-glucoside (SAG) and the glucose ester of SA (SGE) (Vlot et al., 2009; Kobayashi et al., 2020). Several studies expanded the scope of functioning of *SGT1* in plants to include pathogen resistance and mediation of SA production (Song et al., 2007; Hu et al., 2021). The *SGT1* gene identified here may therefore serve as crucial elements for the bioengineering of enhanced disease resistance.

Plant innate immunity mainly consists of two layers of defense, pathogen-associated molecular pattern-triggered immunity (PTI) and effector-triggered immunity (ETI), which help host to fight against pathogens during early attack. Mitogen-activated protein kinase (MAPK) cascades play vital roles in both PTI and ETI signaling pathways (Thomma et al., 2011; Meng and Zhang, 2013; Bigeard et al., 2015). The canonical MAPK cascade consists of MAPKs (MPKs), MAPK kinases (MAPKKs/MAP2Ks/MKKs/MEKs), and MAPK kinase kinases (MAPKKKs/MAP3Ks/MEKKs) (Zhang et al., 2006). The *Arabidopsis* genome contains approximately 20 MAPKs, 10 MAPKKs, and 80 MAPKKKs (Colcombet and Hirt, 2008). Of these, MEKK1-MKK4/5-MPK3/6, MAPKKK3/5-MKK4/5-MAPK3/6, and MEKK1-MKK1/2-MPK4 are generally reported to confer resistance against pathogens (Qiu et al., 2008; Galletti et al., 2011; Xu et al., 2016; Su et al., 2017; Sun et al., 2018). In cucumber, there have been 14 MAPK, 6 MAPKK, and 59 MAPKKK genes identified based on sequence (Wang et al., 2015). To date, only TIPK (a homolog of *Arabidopsis* MPK3) was been discovered to participate in the defense of cucumber against pathogens (Shoresh et al., 2006). In our study, *MPK9* (CsGy1G006960), and *MPK20* (CsGy6G022180) were found to be up-regulated in the resistant line, with no changes observed in the susceptible strain upon inoculation with *P. xanthii*. *MPK9* plays a pivotal role in regulating ABA and methyl jasmonate (MeJA) signaling pathways in *Arabidopsis* guard cells (Jammes et al., 2009; Khokon et al., 2015). ABA and MeJA are important defense signals that impart resistance against pathogen attack (Niu et al., 2010; Cao et al., 2011), so the altered expression of these genes suggests that *MPK9* might also work in response to pathogen infection. An *MPK20* homolog found in cucumber was previously reported to participate in the auxin signaling pathway and primary cell wall formation (Persson et al., 2005; Lalonde et al., 2008). In addition, this gene has been demonstrated to mediate resistance to *Fusarium oxysporum* in cotton (Wang C. et al., 2018).

Likewise, many genes related to MAPK cascades, such as *VIP1* (CsGy6G004900), *CAT3* (CsGy6G018080), and *PYL4* (CsGy4G015470 and CsGy5G008430) were induced in the resistant cucumber strain during *P. xanthii* attack. *VIP1*, which encodes a bZip transcription factor, has been identified as the downstream target of MPK3 in Arabidopsis (Djamei et al., 2007). Under pathogen stress, when phosphorylated by MPK3, the VIP1 protein directly induces the *MYB44* stress-response gene (Pitzschke et al., 2009). CAT (catalase) enzymes are H_2_O_2_ scavengers that maintains ROS homeostasis in various stress responses (Du et al., 2008). *Arabidopsis* ABA-induced *CAT3* expression has been shown to be impaired in a *MEK1* mutant (Xing et al., 2007). *PYL*, which encodes a core component of the ABA signaling pathway, has previously been reported to activate a MAPK cascade (Danquah et al., 2015). *PYL* mediates multiple biological processes, including leaf senescence, lateral root growth, dormancy, and stress responses (Zhao et al., 2014, 2016; Lee et al., 2015; Du et al., 2017). Alteration of the expression of these genes in the cucumber plant, therefore, may be important molecular events in the defense against *P. xanthii.*

After activation of defense mechanisms, plants transfer a series of signals to transcription factors (TFs). Then, TF activate or repress the expression of target genes to resist pathogen damage. Our Mapman analysis revealed that the Dof and WRKY TFs are affected by *P. xanthii* in the resistant cucumber but that these TFs were absent in the susceptible line. Dof transcription factors are unique to plants, with 36 members present in cucumber (Wen et al., 2016). Multiple studies have reported participation of Dofs in pathogen resistance pathways *via* the mediating of the expression of target resistance genes, including Sar8.2b, *ACBP3*, and cystatin (Song and Goodman, 2002; Martinez et al., 2005; Zheng et al., 2012). In grape, Dof3 has been described to be positively related to PM resistance (Yu et al., 2019). The up-regulation of Dofs was also documented when cucumber plants were exposed to the pathogen *Pseudoperonospora cubensis*. Thus, Dofs tend to be regarded as positive regulators in plant defenses against biotic stresses. Here, we found 3 Dof-coding genes (CsGy5G000240 and CsGy6G034890) to be weakly up-regulated and 3 (CsGy3G036390, CsGy6G014450, and CsGy1G005390) significantly down-regulated at 6 hpi in the resistant cucumber. Hence, the link between Dofs and host defense responses needs to be further examined.

The other relevant type of TF, the WRKY, forms a large family that is well-characterized in plants (Ulker and Somssich, 2004). Numerous studies have proposed a crucial role of WRKY in plant defense (Eulgem and Somssich, 2007; Amorim et al., 2017; Wani et al., 2021). Examples of WRKY that play roles in pathogen resistance response include *Triticum aestivum* WRKY19 in the defense against *Puccinia striiformis*, *Fragaria* × *ananassa* WRKY50 in the defense against *Botrytis cinerea*, *Oryza sativa* WRKY6 in the defense against *Xanthomonas oryzae*, and *Hordeum vulgare* WRKY6 in the defense against *Pyrenophora teres* (Ma et al., 2021; Tamang et al., 2021; Im et al., 2022; Nobori, 2022). Cucumber contains 61 WRKY that are classified into 3 main groups (I, II, and III) (Chen et al., 2020). Our data showed that the resistant cucumber exposed to *P. xanthii* stress obviously up-regulated the expression of *WRKY31* (CsGy7G003250). Cucumber *WRKY31* belongs to Group IIb gene that showed orthologous relationship with *Arabidopsis WRKY6*. The protein coded by *WRKY6* was reported to participate in pathogen defense by modulating pathogen defense-associated PR1 promoter activity (Robatzek and Somssich, 2002).

Stress-responsive genes have also been found to have indispensable roles in plant defenses. According to our WGCNA analysis, *GLPK*, *ILK1*, *EIN2*, *BCDH*β*1*, and *RGGA* were found to be highly associated with the *P. xanthii* stress response. Glycerol kinase phosphorylates glycerol to glycerol-3-phosphate (G3P), which is well known as a critical regulator of plant systemic immunity (Chanda et al., 2011). An *Arabidopsis* glycerol kinase gene (*GLI1*) was found to act as a positive modulator of defense toward bacterial and fungal diseases (Kang et al., 2003; Yang et al., 2013). In addition, *GLI1* was found to be induced following PM infections in *Triticum aestivum* (Li et al., 2020). Similarly, we found that the cucumber orthologs of *GLI1* was up-regulated in the resistant strain after *P. xanthii* treatment. *ILK1* encodes a Raf-like MAPKKKs, as it is the major regulator of cell proliferation growth and immune responses in animals (Hannigan et al., 2011). In terms of molecular function in plants, *ILK1* has been identified to control pathogen resistance (Brauer et al., 2016). *EIN2* is also essential for plant immune response, as *ein2* mutants confer sensitivity toward the pathogen fungus (Thomma et al., 1999; Sun et al., 2017). Information regarding *RGGA* and *BCDH* β*1* have linked these genes to tolerance of osmotic and nutrient stresses, respectively (Ambrosone et al., 2015). However, there has been little work on these two genes in the context of plant defense against biotic stresses.

To summarize, this paper showcases key molecular players in plant defense against fungal infection by comparing physiological and gene expression responses in the PM-resistant cucumber strain B21-a-2-1-2 to those of the PM-susceptible strain B21-a-2-2-2 in early stages of infection with *P. xanthii*. After 6 h of *P. xanthii* stress, 472 DEGs (193 up-regulated and 279 down-regulated) were identified in the susceptible line, while 2,473 DEGs (1,242 up-regulated and 1,231 down-regulated) were identified in the resistant line, indicating that the resistant line can rapidly instigate resistance events to fight against pathogen attack. These DEGs were grouped by their functional annotation through GO, KEGG, and Mapman analyses. Genes related to the cell wall and to responses to pathogen and hormone were commonly enriched in both lines under *P. xanthii* stress. Importantly, DEGs related to SA signaling, MAPK signaling and WRKY and Dof transcription factors were found to be involved in the regulation of defense against *P. xanthii* in the resistant cucumber, but these genes were not identified as DEGs in the susceptible line. Meanwhile, 5 hub genes, including *GLPK*, *ILK1*, *EIN2*, *BCDH*β*1*, and *RGGA*, were found to be highly associated with the plant immune response by WGCNA analysis. Our study provides valuable information on the early immune response and aids the understanding of the resistance mechanisms of cucumber to *P. xanthii* stress.

## Data Availability Statement

The data presented in the study are deposited in the NCBI SRA repository, accession number PRJNA816625 (https://www.ncbi.nlm.nih.gov/sra/PRJNA816625).

## Author Contributions

LC and HF conceived and designed the research. TS provided plant materials. YBY and ZM conducted the experiments. XM analyzed the data and wrote the manuscript. YY and NC revised the manuscript. All authors read and approved the manuscript.

## Conflict of Interest

The authors declare that the research was conducted in the absence of any commercial or financial relationships that could be construed as a potential conflict of interest.

## Publisher’s Note

All claims expressed in this article are solely those of the authors and do not necessarily represent those of their affiliated organizations, or those of the publisher, the editors and the reviewers. Any product that may be evaluated in this article, or claim that may be made by its manufacturer, is not guaranteed or endorsed by the publisher.

## Footnotes

1
http://www.cucurbitgenomics.org/organism/16

2
https://daehwankimlab.github.io/hisat2/

3
http://www.geneontology.org/

4
http://www.genome.jp/kegg/

5
https://mapman.gabipd.org/

## Supplementary Material

Supplementary Figure 1 Morphological changes and qRT-PCR detection of *P. xanthii* infection of susceptible strain B21-a-2-2-2 and resistant strain B21-a-2-1-2 for 7 days.

Supplementary Figure 2 A correlation coefficient analysis of samples.

Supplementary Figure 3 Principal components analysis of samples.

Supplementary Figure 4 Volcano diagram analysis of DEGs.

Supplementary Figure 5 Directed acyclic graph for GO terms of biology process in S0h vs. S6h.

Supplementary Figure 6 Directed acyclic graph for GO terms of cellular component in S0h vs. S6h.

Supplementary Figure 7 Directed acyclic graph for GO terms of molecular function in S0h v. S6h.

Supplementary Figure 8 Directed acyclic graph for GO terms of biology process in R0h vs. R6h.

Supplementary Figure 9 Directed acyclic graph for GO terms of cellular component in R0h vs. R6h.

Supplementary Figure 10 Directed acyclic graph for GO terms of molecular function in R0h vs. R6h.

Supplementary Table 1 List of primers used in this study.

Supplementary Table 2 Principal component analysis of samples.

Supplementary Table 3 List of DEGs in S0h vs. R0h comparison.

Supplementary Table 4 List of DEGs in S0h vs. S6h comparison.

Supplementary Table 5 List of DEGs in R0h vs. R6h comparison.

Supplementary Table 6 List of DEGs identified as the same expression trend in S0h vs. S6h, R0h vs. R6h, and S6h vs. R6h comparisons.
