# Supplementary figures and images for: Transcriptome Sequence Analysis of the Defense Responses of Resistant and Susceptible Cucumber Strains to Podosphaera xanthii
Source: Front Plant Sci. 2022 May 12;13:872218. doi: 10.3389/fpls.2022.872218 (PMC9134894; doi:10.3389/fpls.2022.872218)

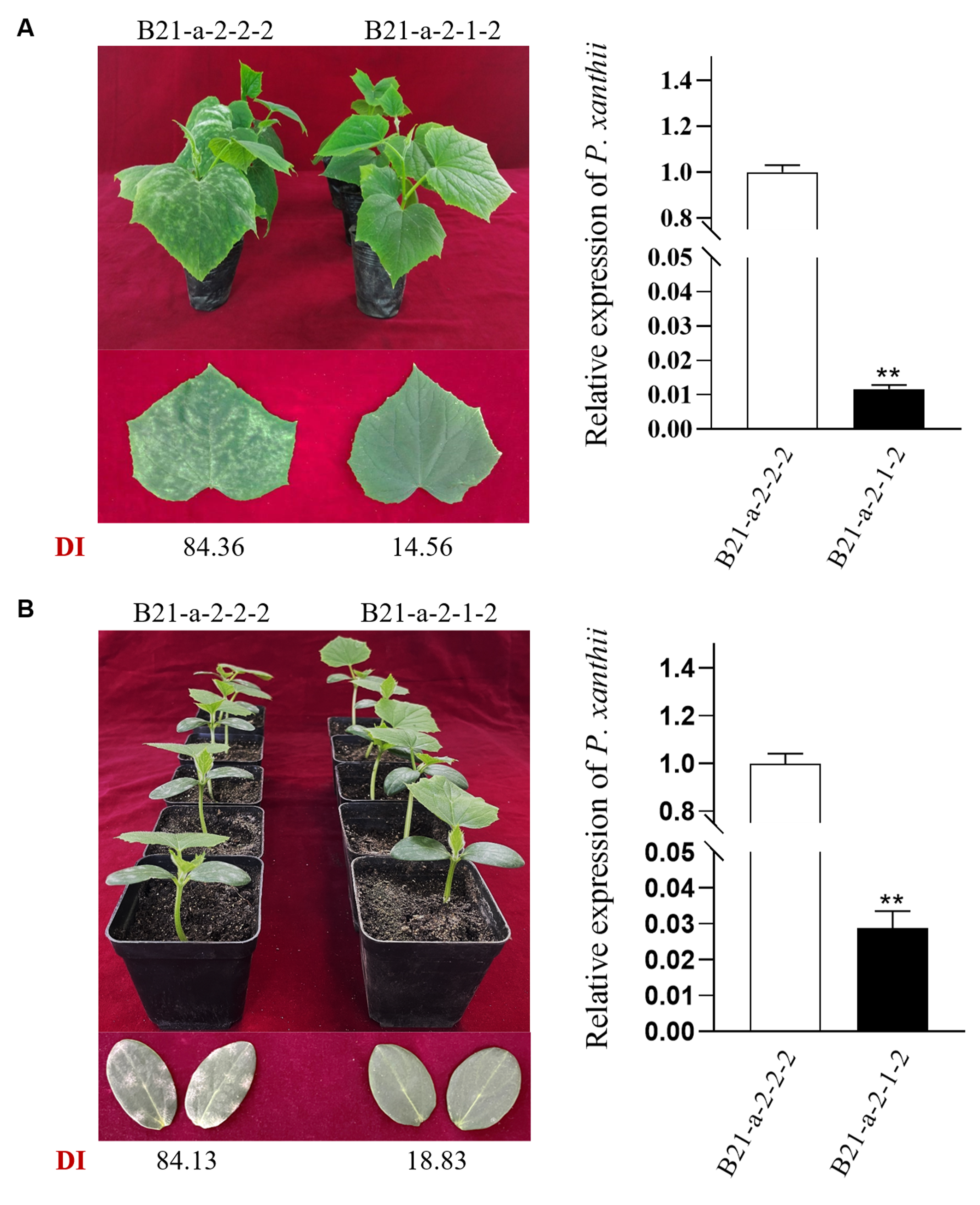

Supplement: Supplementary Figure 1 — Morphological changes and qRT-PCR detection of P. xanthii infection of susceptible strain B21-a-2-2-2 and resistant strain B21-a-2-1-2 for 7 days. [file Image_1.TIF]

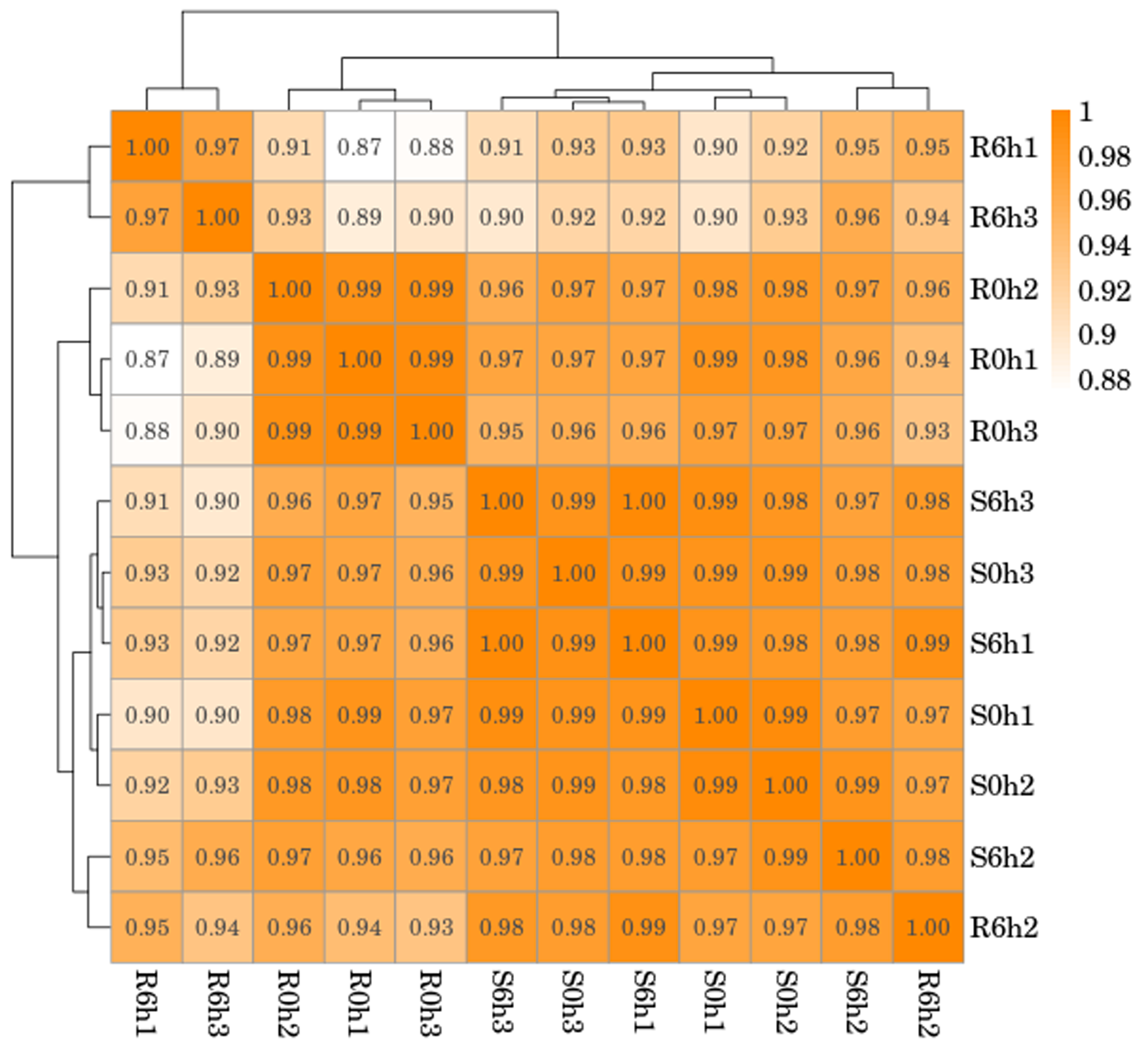

Supplement: Supplementary Figure 2 — A correlation coefficient analysis of samples. [file Image_2.TIF]

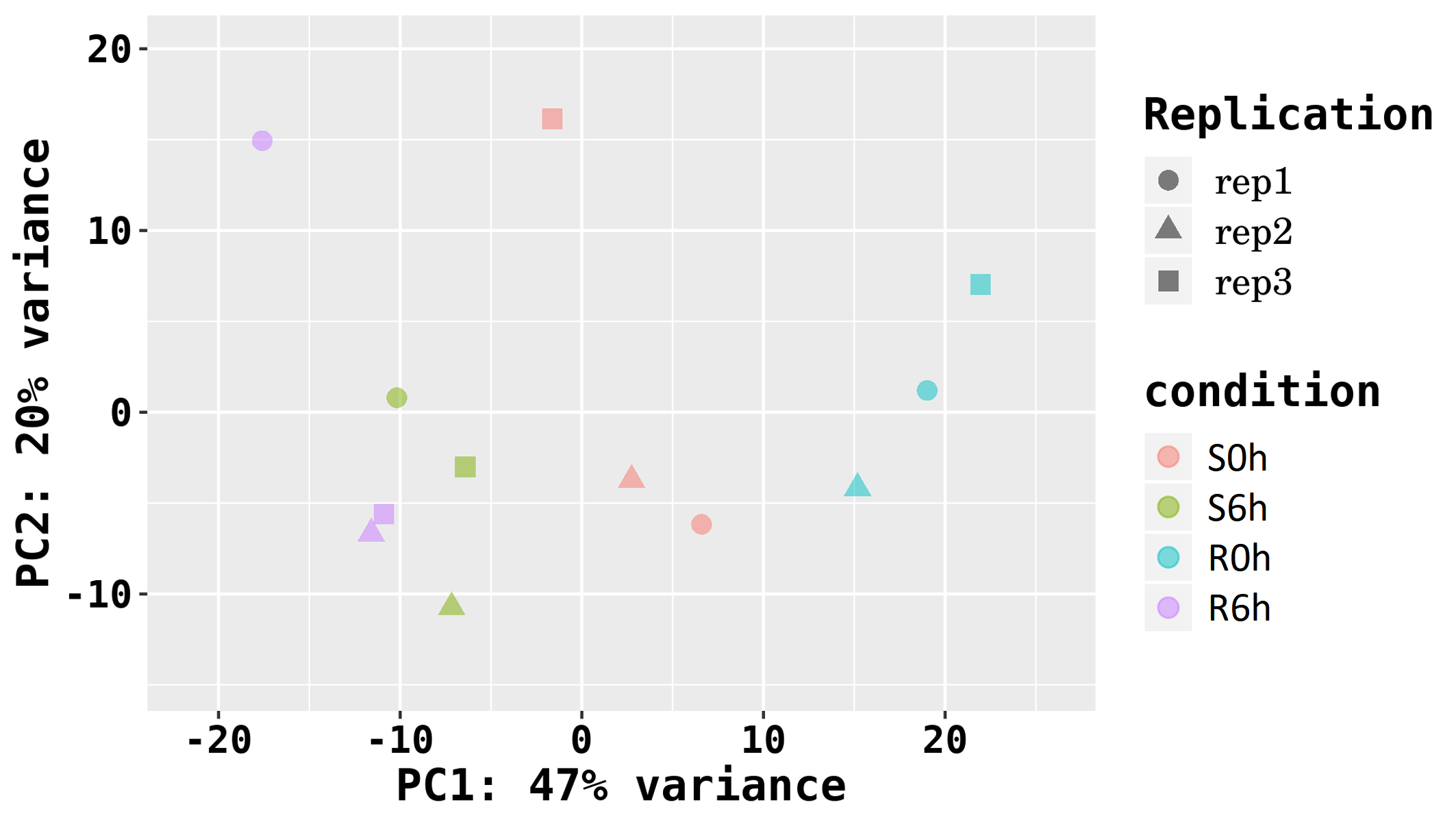

Supplement: Supplementary Figure 3 — Principal components analysis of samples. [file Image_3.TIF]

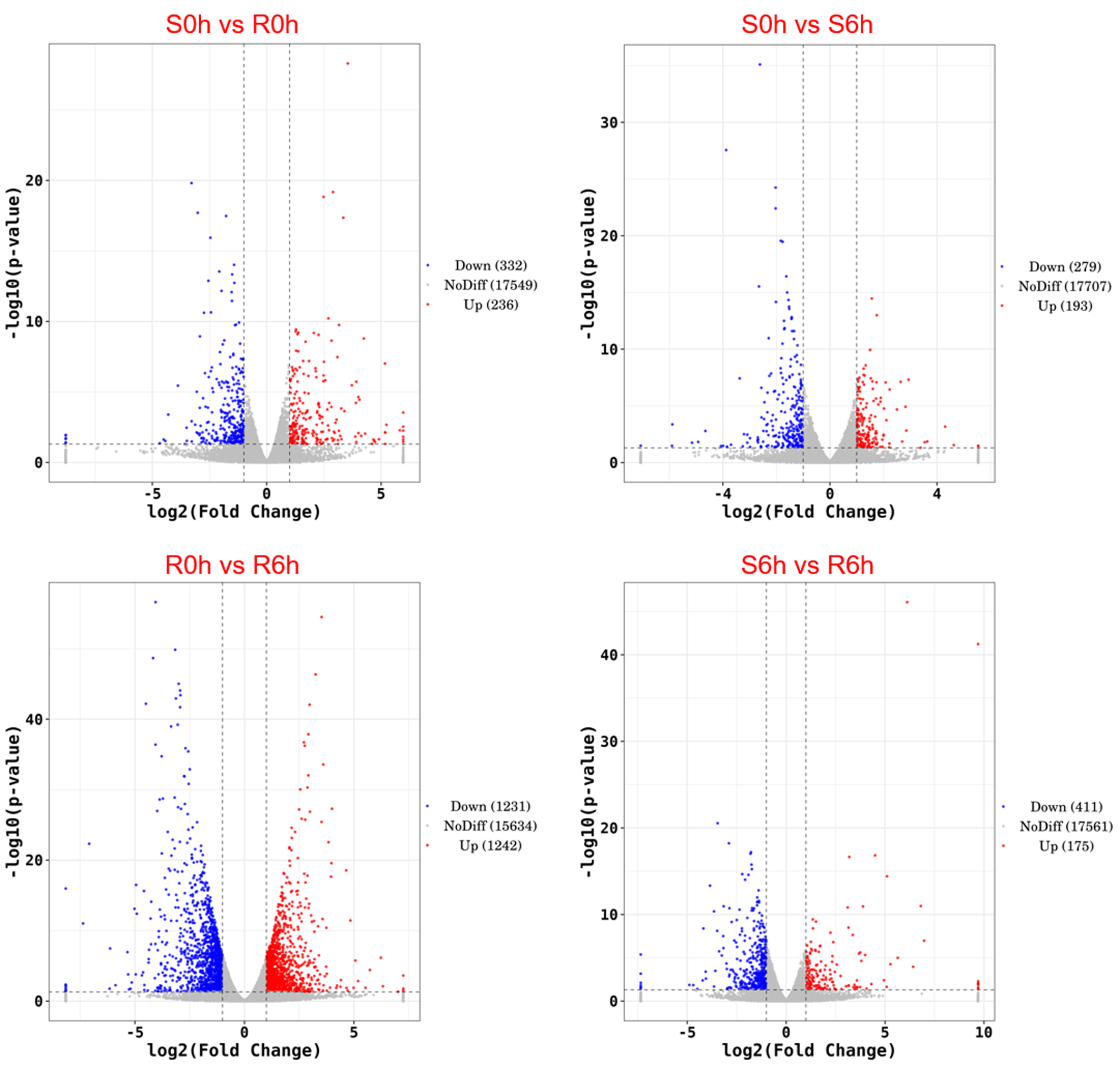

Supplement: Supplementary Figure 4 — Volcano diagram analysis of DEGs. [file Image_4.TIF]

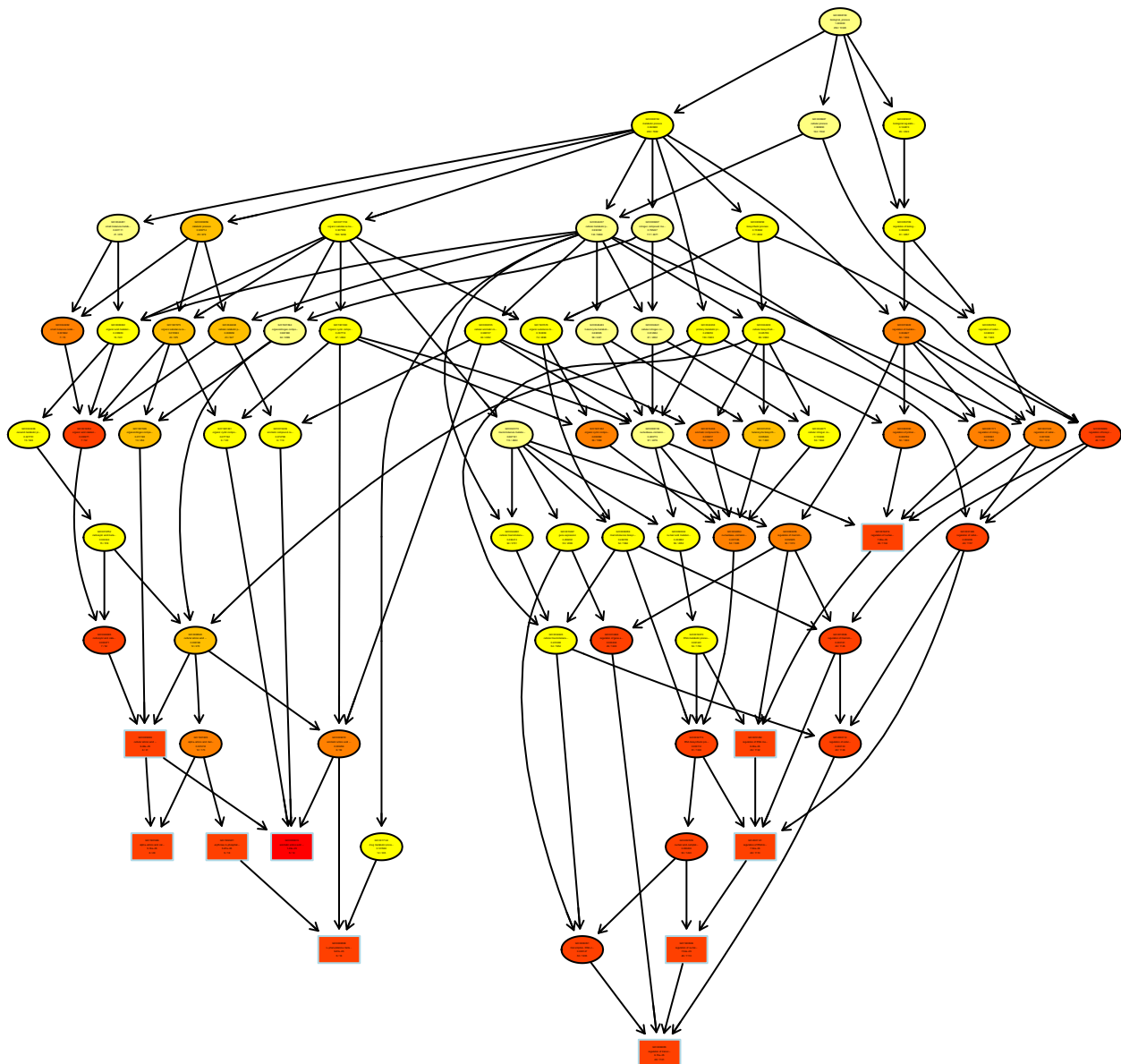

Supplement: Supplementary Figure 5 — Directed acyclic graph for GO terms of biology process in S0h vs. S6h. [file Image_5.PDF]

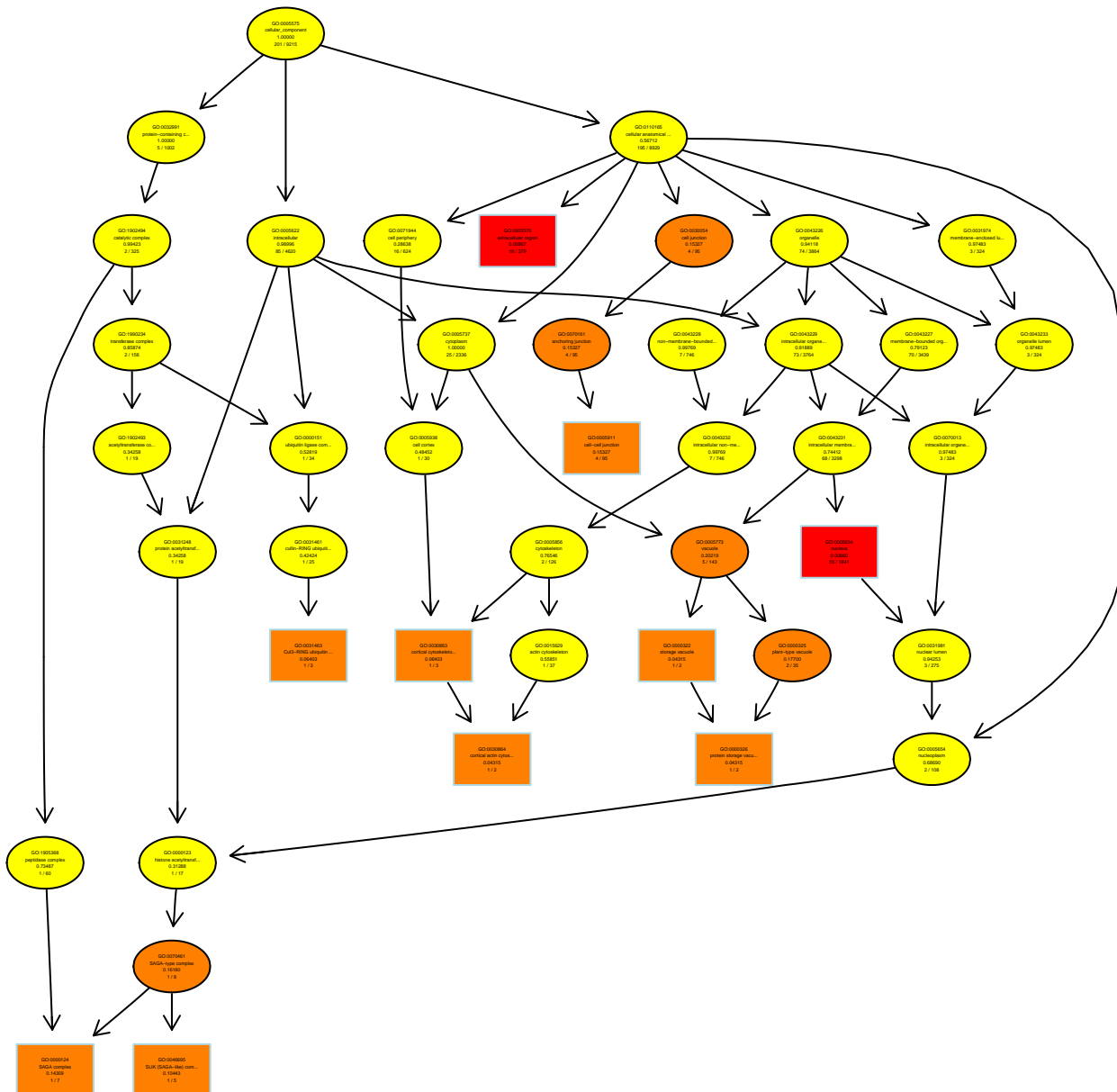

Supplement: Supplementary Figure 6 — Directed acyclic graph for GO terms of cellular component in S0h vs. S6h. [file Image_6.PDF]

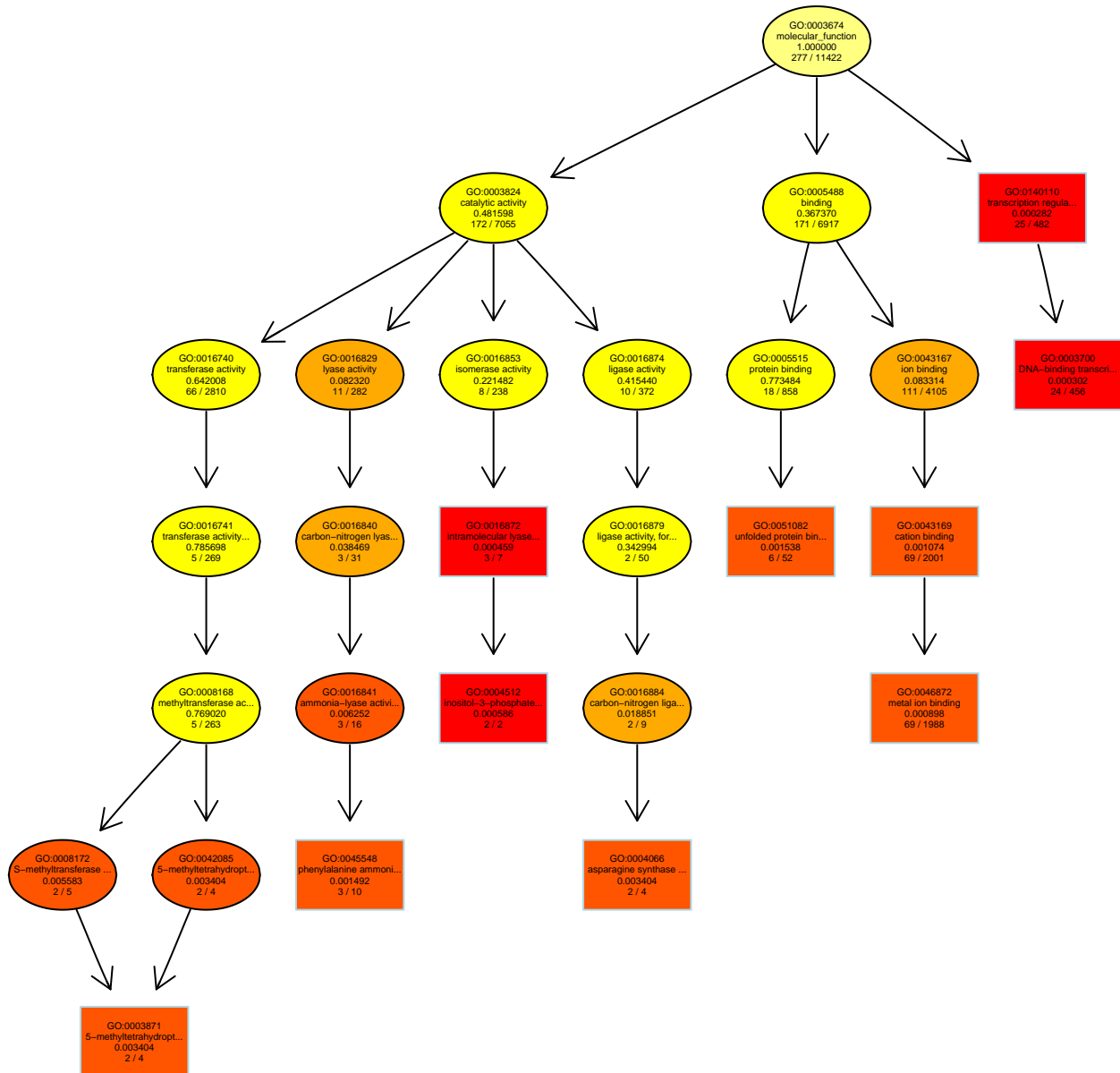

Supplement: Supplementary Figure 7 — Directed acyclic graph for GO terms of molecular function in S0h v. S6h. [file Image_7.PDF]

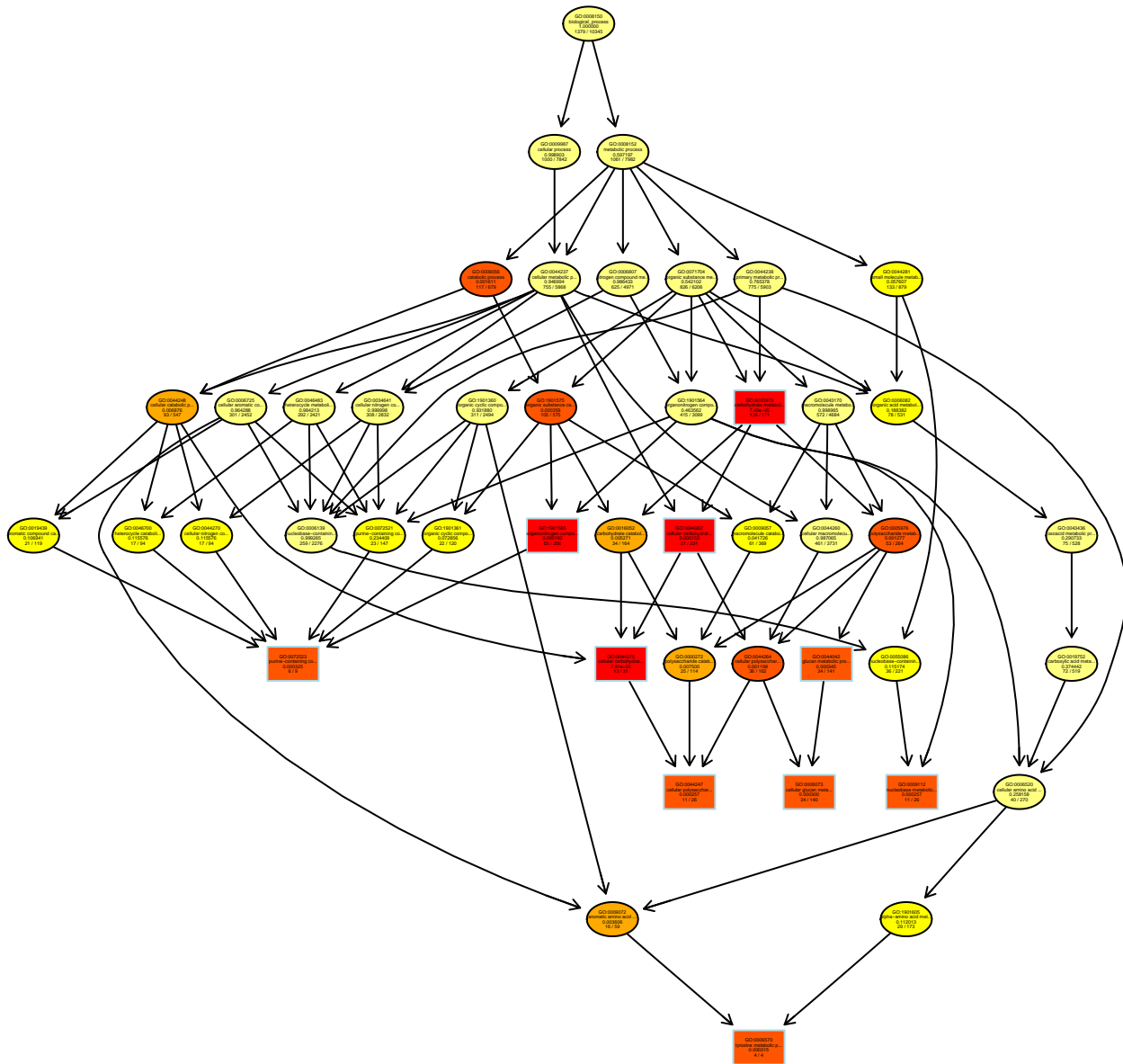

Supplement: Supplementary Figure 8 — Directed acyclic graph for GO terms of biology process in R0h vs. R6h. [file Image_8.PDF]

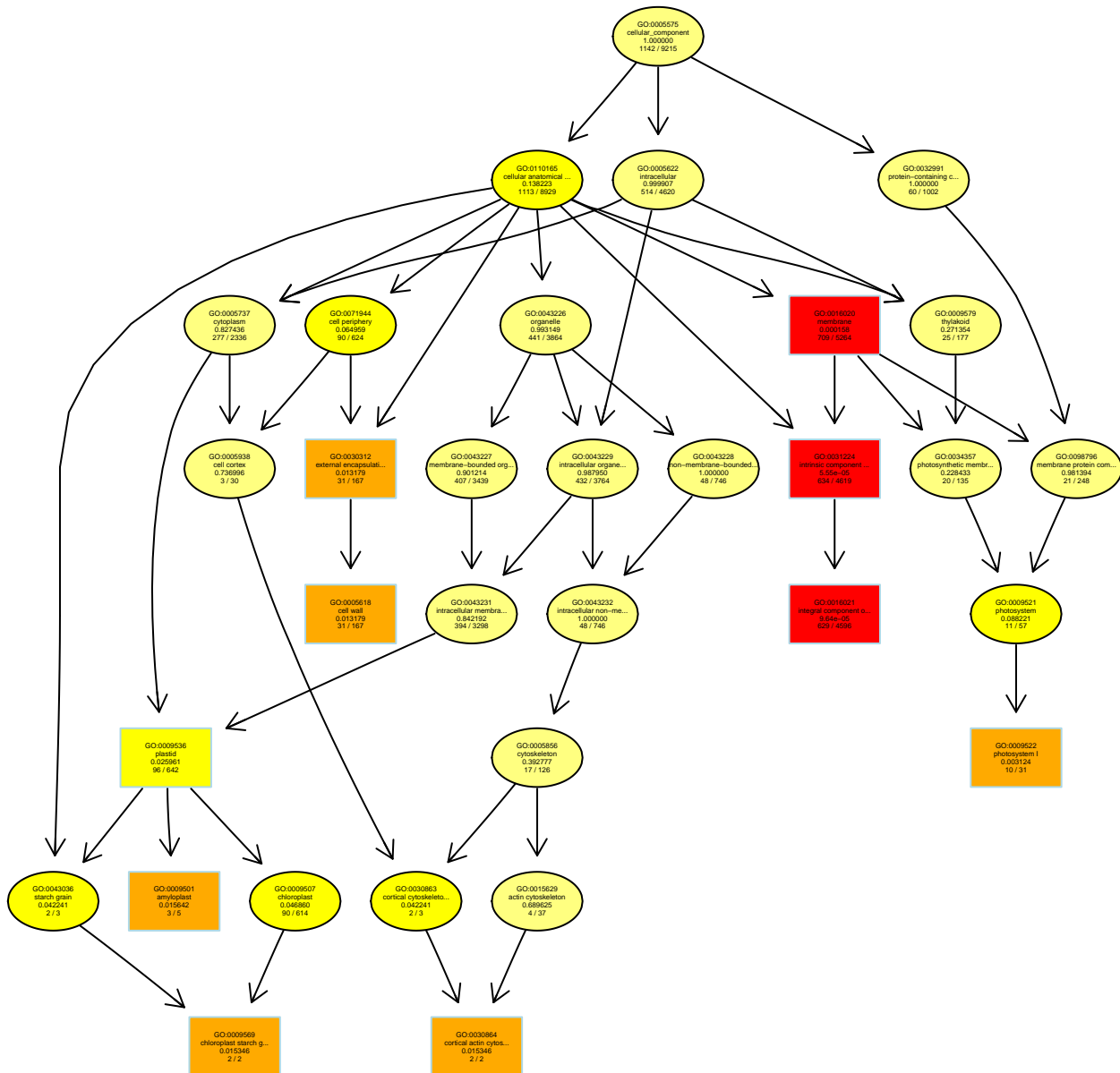

Supplement: Supplementary Figure 9 — Directed acyclic graph for GO terms of cellular component in R0h vs. R6h. [file Image_9.PDF]

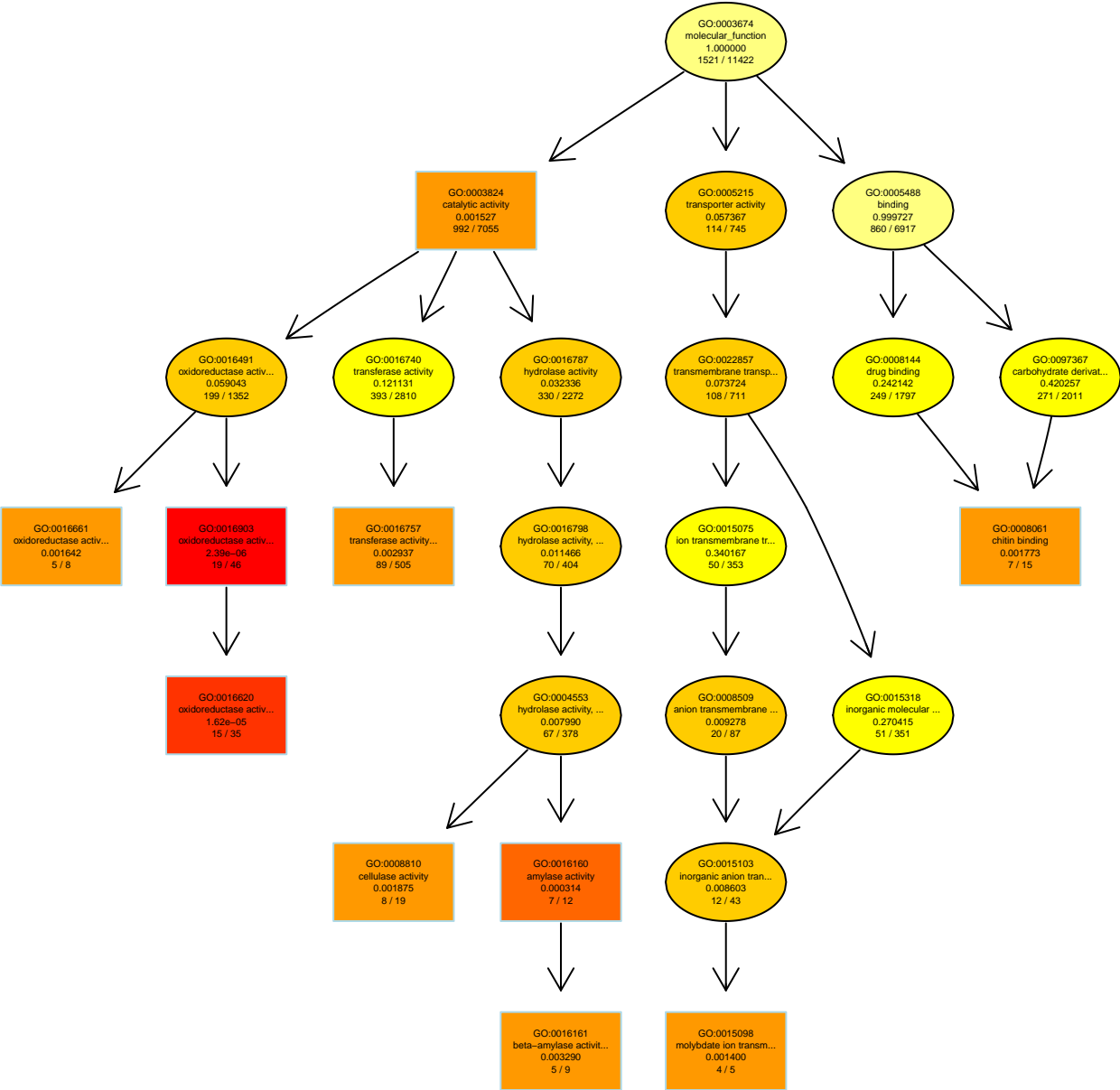

Supplement: Supplementary Figure 10 — Directed acyclic graph for GO terms of molecular function in R0h vs. R6h. [file Image_10.PDF]
