# Supplementary material for: Transcriptome Sequence Analysis of the Defense Responses of Resistant and Susceptible Cucumber Strains to Podosphaera xanthii
Source: Front Plant Sci. 2022 May 12;13:872218. doi: 10.3389/fpls.2022.872218 (PMC9134894; doi:10.3389/fpls.2022.872218)
Supplement: Supplementary Table 1 — List of primers used in this study. [file Table_1.DOCX]

**Table S1** List of primers used in this study

| Name | Gene ID | Primer |
| --- | --- | --- |
| *VIP1* | CsGy6G004900 | F: 5'-AGTACAGCGACTTAAGATAGCC-3'  R: 5'-CTAAAGCATGTGGTTAAGCTCC-3' |
| *MAPK20* | CsGy6G022180 | F: 5'-CTTTCTTTATGGCACGTGTAGG-3'  R: 5'-CAGTGTTTCTATGGGTAGTGGT-3' |
| *CAT3* | CsGy6G018080 | F: 5'-CACAACTTGGTTTGTTCAAACG-3'  R: 5'-AATCTTCTAACAGGATTGGGCC-3' |
| *MPK9* | CsGy1G006960 | F: 5'-TGTATCTGATGCCATACGGATC-3'  R: 5'-CTTTGAATTCTCGTTGTGAGGG-3' |
| *POX 2-like* | CsGy4G012840 | F: 5'-AGCAGAACAGCCAATAAAACAG-3'  R: 5'-GAAGTTCTTGCCTGTAAGTTGG-3' |
| *CSLA9* | CsGy6G024620 | F: 5'-GATTATCATTTCACCGTGGAGC-3'  R: 5'-CCAAGTCCATATCTTCGACTGT-3' |
| *EXP-A4* | CsGy1G022650 | F: 5'-CGGCAAGAATTTTAGGGTTTGA-3'  R: 5'-TAATAATGTAGGACTGTGGGCC-3' |
| *XTH* | CsGy6G019970 | F: 5'-GCATATTACCGCAACTTCAACT-3'  R: 5'-GTAATGGTGGTGATTGCATTCA-3' |
| *CDF3* | CsGy1G005390 | F: 5'-GGCTCTGTTTTAAACCTTGGAG-3'  R: 5'-GGAAGACAAACTCGAGCTTTTT-3' |
| *bZip34-like* | CsGy2G025690 | F: 5'-ACCAACAGCGACTACTTCTAAA-3'  R: 5'-TGTTGTCGATGATACAATTGCC-3' |
| *bZip34* | CsGy4G021500 | F: 5'-CATGAAGAATGAACCTGCTGAC-3'  R: 5'-CTCTCGATCTTTGTGCTGATTG-3' |
| *Dof 1.7-like* | CsGy1G006100 | F: 5'-CCCGGAAATCGAAGAAGAATTC-3'  R: 5'-GAAATTCAACCACGAACTTCCA-3' |
| *Actin* | CsGy6G026130 | F: 5'-TCGTGCTGGATTCTGGTG-3'  R: 5'-GGCAGTGGTGGTGAACAT-3' |
| *S. fuliginea-ITS* | MW939431 | F: 5'-ATTACTGAGCGCGAGGCCCCG-3'  R: 5'-CGCGAGATACATGACTACGC-3' |
