# Supplementary material for: Transcriptome Sequence Analysis of the Defense Responses of Resistant and Susceptible Cucumber Strains to Podosphaera xanthii
Source: Front Plant Sci. 2022 May 12;13:872218. doi: 10.3389/fpls.2022.872218 (PMC9134894; doi:10.3389/fpls.2022.872218)
Supplement: Supplementary Table 2 — Principal component analysis of samples. [file Table_2.DOCX]

**Table S2** Principal components analysis of samples

| **Sample** | **Replication** | **PC1** | **PC2** |
| --- | --- | --- | --- |
| S0h1 | rep1 | 6.592691567 | -6.169774148 |
| S0h2 | rep2 | 2.742526893 | -3.726261452 |
| S0h3 | rep3 | -1.632036406 | 16.14220842 |
| R0h1 | rep1 | 19.0122686 | 1.195046545 |
| R0h2 | rep2 | 15.18223692 | -4.172441182 |
| R0h3 | rep3 | 21.94607899 | 7.025018487 |
| S6h1 | rep1 | -10.18221347 | 0.796560581 |
| S6h2 | rep2 | -7.163800313 | -10.73848459 |
| S6h3 | rep3 | -6.419514114 | -3.003793382 |
| R6h1 | rep1 | -17.59691947 | 14.9414794 |
| R6h2 | rep2 | -11.59243265 | -6.677874215 |
| R6h3 | rep3 | -10.88888654 | -5.611684464 |
