# Supplementary material for: Transcriptome Sequence Analysis of the Defense Responses of Resistant and Susceptible Cucumber Strains to Podosphaera xanthii
Source: Front Plant Sci. 2022 May 12;13:872218. doi: 10.3389/fpls.2022.872218 (PMC9134894; doi:10.3389/fpls.2022.872218)
Supplement: Supplementary Table 6 — List of DEGs identified as the same expression trend in S0h vs. S6h, R0h vs. R6h, and S6h vs. R6h comparisons. [file Table_6.DOCX]

**Table S6** List of DEGs identified as the same expression trend in S0h vs S6h, R0h vs R6h and S6h vs R6h comparisons

| **Gene** | **Log_2_ Fold Change** | | | **Description** |
| --- | --- | --- | --- | --- |
|  | **S0h vs S6h** | **R0h vs R6h** | **S6h vs R6h** |  |
| **Up-regulated** | | | | |
| CsGy4G012840 | 1.26 | 2.93 | 1.18 | peroxidase 2-like |
| CsGy2G000840 | 2.23 | 3.97 | 1.08 | E3 ubiquitin-protein ligase RNF115-like |
| CsGy1G010260 | 1.54 | 4.84 | 1.07 | caffeoyl shikimate esterase-like |
| CsGy6G002460 | 1.06 | 2.37 | 1.88 | glycine-rich cell wall structural protein 1.8-like |
| CsGy6G023580 | 1.11 | 1.14 | 3.37 | calcium-dependent protein kinase 8-like |
| CsGy6G006640 | 1.02 | 1.88 | 1.65 | uncharacterized protein |
| CsGy6G008130 | 1.60 | 2.08 | 1.13 | major pollen allergen Ole e 6 |
| CsGy3G004100 | 1.23 | 2.53 | 1.21 | beta-amylase |
| CsGy3G016890 | 1.08 | 6.23 | 1.27 | uncharacterized protein |
| **Down-regulated** | | | | |
| CsGy1G005110 | -1.83 | -3.63 | -1.75 | protein REVEILLE 2-like |
| CsGy5G013560 | -1.60 | -2.90 | -1.32 | protein REVEILLE 1-like isoform X1 |
| CsGy4G009470 | -1.55 | -4.26 | -2.24 | zinc finger protein CONSTANS-LIKE 2 |
| CsGy1G022650 | -1.72 | -3.54 | -1.21 | expansin-A4-like |
| CsGy5G007310 | -1.77 | -4.50 | -1.45 | ultraviolet-B receptor UVR8 isoform X2 |
| CsGy5G005700 | -1.31 | -4.95 | -1.51 | glycerol-3-phosphate transporter |
| CsGy2G007940 | -1.48 | -3.35 | -2.08 | GDSL esterase/lipase |
| CsGy3G030080 | -1.09 | -3.03 | -1.46 | haloacid dehalogenase-like hydrolase (HAD) superfamily protein |
| CsGy3G030630 | -1.08 | -3.81 | -2.36 | protein REVEILLE 8-like isoform X2 |
| CsGy4G005520 | -1.07 | -2.90 | -1.54 | B-box zinc finger protein 18-like |
| CsGy4G021000 | -1.25 | -2.73 | -1.24 | bidirectional sugar transporter SWEET |
| CsGy3G027980 | -1.01 | -2.27 | -1.25 | 4-coumarate--CoA ligase 1-like |
| CsGy1G025950 | -2.45 | -3.62 | -2.14 | root phototropism protein 3-like isoform X1 |
| CsGy3G000860 | -1.08 | -2.14 | -1.06 | F-box/kelch-repeat protein SKIP25 |
| CsGy6G006600 | -1.68 | -4.26 | -2.31 | dirigent protein |
| CsGy6G024590 | -2.02 | -3.87 | -1.07 | glucomannan 4-beta-mannosyltransferase 9-like |
| CsGy3G038880 | -1.11 | -5.02 | -3.48 | oxygen-dependent choline dehydrogenase |
| CsGy3G027100 | -1.15 | -2.32 | -1.03 | boron transporter 1 |
| CsGy6G018540 | -1.45 | -3.72 | -1.35 | ATP-citrate synthase beta chain protein 1 |
| CsGy7G013600 | -1.22 | -4.06 | -1.56 | putative lipid-transfer protein DIR1 |
| CsGy3G012350 | -1.76 | -3.86 | -1.51 | DNA repair helicase rad5,16, putative |
| CsGy1G020180 | -1.39 | -2.95 | -1.46 | probable receptor-like serine/threonine-protein kinase At5g57670 |
| CsGy3G017310 | -1.38 | -4.06 | -1.96 | beta-carotene hydroxylase |
| CsGy6G010650 | -1.10 | -3.28 | -1.80 | expansin-B3 |
| CsGy2G003100 | -1.03 | -2.67 | -1.02 | auxin-responsive protein |
| CsGy2G017960 | -1.56 | -4.17 | -2.32 | transcription factor MYB1R1-like |
| CsGy6G012760 | -1.08 | -3.91 | -2.58 | hypothetical protein |
